# *SYK* Allelic Loss and the Role of Syk-Regulated Genes in Breast Cancer Survival

**DOI:** 10.1371/journal.pone.0087610

**Published:** 2014-02-11

**Authors:** Jan Blancato, Ashley Graves, Banafsheh Rashidi, Maria Moroni, Leopold Tchobe, Metin Ozdemirli, Bhaskar Kallakury, Kepher H. Makambi, Catalin Marian, Susette C. Mueller

**Affiliations:** 1 Department of Oncology, Lombardi Comprehensive Cancer Center, Georgetown University Medical Center, Washington, D. C., United States of America; 2 Department of Pathology, Georgetown University Medical Center, Washington, D. C., United States of America; 3 Armed Forces Radiobiology Research Institute, Uniformed Services University of the Health Sciences, Bethesda, Maryland, United States of America; 4 University of the District of Columbia/Lombardi Comprehensive Cancer Center Partnership, Washington, D. C., United States of America; 5 Department of Biostatistics and Bioinformatics, Georgetown University Medical Center, Washington, D. C., United States of America; 6 Ohio State University Comprehensive Cancer Center, The Ohio State University, Columbus, Ohio, United States of America; 7 Biochemistry Department, University of Medicine and Pharmacy, “Victor Babes”, Timisoara, Romania; The University of Hong Kong, China

## Abstract

Heterozygotic loss of *SYK*, a non-receptor tyrosine kinase, gives rise to mouse mammary tumor formation where Syk protein levels are reduced by about half; loss of *SYK* mRNA is correlated with invasive cell behavior in *in vitro* models; and *SYK* loss has been correlated with distant metastases in patients. Here, allelic loss of the *SYK* gene was explored in breast ductal carcinoma in situ (DCIS) using fluorescence *in situ* hybridization and pyrosequencing, respectively, and in infiltrating ductal carcinoma (IDC) using genomic data from The Cancer Genome Atlas (TCGA). Allelic loss was present in a subset of DCIS cases where adjacent IDC was present. *SYK* copy number loss was found in about 26% of 1002 total breast cancer cases and 30% of IDC cases. Quantitative immunofluorescence revealed Syk protein to be six-fold higher in infiltrating immune cells compared with epithelial cells. This difference distorted tumor cell mRNA and protein levels in extracts. 20% of 1002 IDC cases contained elevated immune cell infiltration as estimated by elevated immune-specific mRNAs. In cases without immune cell infiltration, loss of *SYK* copy number was associated with a significant reduction of *SYK* mRNA. Here we define a 55 Gene Set consisting of Syk interacting, motility- and invasion-related genes. We found that overall survival was significantly reduced in IDC and Luminal A+B cases where copy number and mutations of these 55 genes were affected (Kaplan-Meier, Logrank test p-value 0.007141 and Logrank test p-value 0.001198, respectively). We conclude that reduction in Syk expression and contributions of genomic instability to copy number and mutations in the 55 Syk interacting genes significantly contribute to poorer overall patient survival. A closer examination of the role of Syk interacting motility and invasion genes and their prognostic and/or causative association with metastatic disease and patient outcome is warranted.

## Introduction

Syk (spleen tyrosine kinase), a 72 kD tyrosine kinase, plays diverse and complicated roles in immunity as well as in epithelial cell biology and is required for activation of immune, integrin, and a growing number of other cell surface receptors [1]. In normal or cancerous mammary epithelial cells, the presence of Syk suppresses malignant growth characteristics including proliferation and invasive cell migration and its loss induces invasion and metastasis [2], [3]. Previously, in patient samples, we demonstrated a loss of *SYK* mRNA in normal tissue adjacent to breast cancer, and further decreases in ductal carcinoma in situ (DCIS) and intraductal carcinoma (IDC) compared with normal or benign tissues where no cancer was detected [4]. Others have validated the association with Syk loss and breast cancer progression ([5] and references therein).

*SYK* loss has been attributed to hypermethylation of the promoter in breast cancer tissues and its loss is associated with increased cellular invasiveness [6], [7]. Hypermethylation of the *SYK* promoter is associated with lower *SYK* mRNA and poor prognosis and metastasis in various cancers including breast, lung, pancreatic, urinary bladder cancers, mesothelioma, and melanoma; *in vitro* experiments confirm that re-expression of *SYK* by transfection or inhibitors of hypermethylation reverses the invasive and metastatic phenotype ([7]–[15] and for review [5]). *SYK* hypermethylation was noted in 45% of DCIS but only 5% of hyperplasia, thus, hypermethylation in DCIS tissues occurs prior to the development of invasive disease [7]. It was inferred that *SYK* loss might contribute to the development of invasive breast cancer. Interestingly, *SYK* mRNA loss in postoperative lung metastases was noted although not directly validated post-surgery in an orthotopic mouse model compared with lung metastases of control mice whose primary tumor was not resected [16]. Recently, several studies have identified SNPs and somatic mutations of *SYK* associated with breast cancer [17], [18].

Examination of heterozygotic *SYK* knockdown mice and derivatives of these mice revealed that loss of a single *SYK* allele was sufficient to reduce Syk protein levels by about half [3]. Syk loss led to accelerated proliferation and ductal outgrowth during puberty and mammary tumor formation *in vivo*, although the causality of mammary-specific *SYK* loss was not determined [3]. *In vitro*, enhanced cell proliferation and invasion were detected in mouse epithelial cells isolated from heterozygotic knockdown mice [3]. In the same study, transient or stable Syk knockdown in a non-transformed human epithelial cell line MCF10A had the same effects; proliferation and invasion were enhanced [3]. These data taken together also significantly strengthen the argument that Syk is a potent breast cancer tumor and metastasis suppressor. Thus, *SYK* loss results in dramatic effects upon epithelial cell function, promoting proliferative and/or invasive behaviors.

*SYK* is located on human chromosome 9q22.2 and interestingly, allelic loss on chromosome 9q22 is associated with lymph node metastasis in primary breast cancer [19]. However, in four breast cancer cell lines, Coopman and colleagues found no evidence for alterations in *SYK* DNA by Southern analysis [2]. To determine whether allelic loss of *SYK* might be associated with breast cancer invasion and progression in patient samples, we performed fluorescent *in situ* hybridization (FISH) to detect *SYK* alleles in a dual color FISH protocol. We focused on DCIS tissues for analysis with comparison to normal or benign tissues. The rationale for this includes the previous observation that single allelic loss of *SYK* in a mouse model led to enhanced invasion, proliferation, and tumor formation in the mammary gland; Syk knockdown in cells results in an epithelial-to-mesenchymal (EMT)-like transition with enhanced invasiveness; and Syk re-expression in breast cancer cell lines prevents tumor growth and metastasis in mouse models [2], [3], [5]. Thus, *SYK* allelic loss in human DCIS, particularly in combination with hypermethylation and silencing might result in invasive breast disease ultimately leading to metastasis. If so, *SYK* status in DCIS tissues might provide a powerful prognostic tool, identifying women most likely to progress to invasive carcinoma. Many women with DCIS will relapse and progress to IDC [20], [21].

Strikingly, the results of our preliminary study of clinical breast cancer tissues revealed that *SYK* gene loss occurred in 5 out 19 samples of DCIS examined, but in none of 5 normal breast tissues examined. Allelic loss did not occur in DCIS only cases, but rather was exclusively associated with DCIS that was adjacent to IDC. *SYK* loss in IDC was determined in a large breast cancer data set from The Cancer Genome Atlas (TCGA) using cBioPortal tools. Furthermore, genes relating to motility and invasion that we previously demonstrated to be regulated by S*YK* were used to query a TCGA breast study. The results indicated that overall survival was significantly influenced by this gene set (51 genes); prediction of improved overall survival could be further enhanced by the addition of *TP53*, *SRC*, *CTTN*, and *CDH1*, genes that interact with the *SYK* network or Syk directly.

## Materials and Methods

### Tissues

Ethics statement: formalin-fixed, paraffin-embedded breast tissue samples were obtained from the Lombardi Comprehensive Cancer Center (LCCC) Histopathology and Tissue Shared Resource (HTSR) using a Georgetown University approved IRB protocol and adjacent sections were used in a previous study [4]. The relevant Georgetown University Institutional Review Board (IRB) protocol was # 1992-048, “Human Tissue Bank”, B. Kallakury, HTSR, and this protocol has been continuously maintained from 1992 to present date. Written consent was obtained from patients for surgery and excess tissue was banked after diagnostic requirements were met. The patients were all pre-HIPAA. Materials and information from patients were de-identified. A serial section from each tissue sample was stained with hematoxylin and eosin and reviewed by pathologists Drs. Metin Ozdemirli and Bhaskar Kallakury to identify the appropriate tissue areas for FISH and protein analysis.

### Fluorescence in situ Hybridization (FISH) Probe Development

The Bacterial Artificial Chromosome (BAC) clones, RP11367F29, and RP1183L6 which span the *SYK* gene region at 9q22.2, were obtained from the BAC/PAC Resource at Children's Hospital Oakland Research Institute (Oakland, CA) for development of a FISH probe. The DNA clones were grown on agarose, colonies were picked for expansion, and DNA isolated from cultures using a Qiagen QIA/Prep mini kit. A 2% agarose gel was run to quantify the DNA for probe mixing.

The *SYK* DNA clones were labeled via nick translation with Spectrum Orange™ (Vysis, Des Plaines IL.) A chromosome 9, Spectrum Green™ -labeled centromere control probe was obtained from Vysis (Des Plaines, Ill). The labeled BAC clones and control probes were co-hybridized to metaphase preparations of normal lymphocytes and analyzed with fluorescence microscopy to evaluate the FISH signal strength, clarity, and mapping position.

### Tissue FISH

A dual label FISH technique was used [22]. The slides with paraffin-embedded breast tissue samples were incubated on a slide warmer at 60°C for at least 1 hour. The breast tumor tissue sections were deparaffinized with a 10 minute xylene wash at room temperature (18°C to 25°C) and dehydrated twice in 100% ethanol for 5 minutes at room temperature (18°C to 25°C). The pretreatment solution was 10% sodium borohydrite in 2× Saline Sodium Citrate (SSC) for 15 minutes. Protein digestion (25 µg/ml) was performed with Proteinase K (Sigma-Aldrich, St Louis, MO, USA) in 2× SSC at 45°C for 10 minutes. The co-denaturation of the test and control probes and target sequence was performed at 85°C for 10 minutes on a hot plate under a coverslip. Following denaturation, the coverslip edges were sealed with rubber cement and hybridization was carried out at 37°C in a humid chamber for 24 hours overnight. Sixteen to 24 hours later the coverslips were removed and slides were then post washed in 2× SSC at 72°C for 5 minutes. The slides were counterstained with 4′,6-diamidino-2-phenylindole (DAPI). The slides were then viewed with fluorescence microscopy with a 100× objective using filters that allow the probe signals to be visualized. 30 cells were counted. The data were expressed as number of *SYK* signals/number of chromosome 9 signals.

The SYK FISH performed on tumor tissue sections has the advantage over PCR protocols of preserving specimen architecture, allowing the analysis to be focused on neoplastic tissue, without contamination of normal surrounding tissues including immune cells. Also, individual tumor cells can be analyzed for both control and loci of interest. This provides specificity to the analysis and an internal control of the assay. Tissue FISH was performed as a research protocol in our clinical lab and is the approved method for clinical analysis of tissues for diagnosis, prognosis and management by the American College of Medical Genetics (ACMG). The ACMG writes and advised laboratory standards for Clinical Genetics [23].

### Methylation

DNA methylation level was assessed by bisulfite pyrosequencing with the Pyromark MD (QIAGEN) instrument, using the Hs_*SYK*_01_PM PyroMark CpG Assay, a pre-designed assay specific for a CpG rich region within the *SYK* gene promoter available from QIAGEN. Genomic DNA was extracted from tissue dissected off paraffin section slides as previously described [24] and then subjected to bisulfite pyrosequencing. Pyrosequencing is a sequencing-by-synthesis method that quantitatively monitors the real-time incorporation of nucleotides through the enzymatic conversion of released pyrophosphate into a proportional light signal, thus enabling a quantitative measurement of the methylation extent at each CpG site [25]. Briefly, the DNA was treated with sodium bisulfite using the EpiTect Bisulfite Kit (Qiagen) in order to convert all un-methylated cytosine residues to uracil which will be then converted to thymine during PCR reaction. A target region of 137 bp was then amplified by PCR using primers from the Hs_*SYK*_01_PM assay (Qiagen) complementary to the bisulfite-treated DNA sequence, amplifying all states irrespective of methylation status. The reverse primer was biotinylated at its 5′-terminus enabling immobilization on streptavidin-coated beads used to purify and render the PCR product single-stranded (as only one strand is biotinylated). The pyrosequencing primer contained in the assay, complementary to the single-stranded template, was then hybridized to the template, and the pyrosequencing reaction was performed by the sequential addition of single nucleotides in a predefined order. The data were then analyzed using the Pyro Q-CpGT Software (Qiagen Inc., Valencia, CA) by calculating the percent methylation for each CpG site in the sequence context.

The assay used herein measured the methylation levels at four CG sites and the mean methylation level was used as a measure of *SYK* gene promoter methylation. In addition to negative controls, we included in the analysis DNA samples from the Human control DNA set (Qiagen) which contains both bisulfite-converted methylated and unmethylated DNA and unconverted unmethylated normal human DNA). The mean methylation level of the control DNAs were 2.32% for the non-methylated DNA, 70.54% for the methylated DNA, and 4.70% for the normal human DNA.

### Immunofluorescence Microscopy

For double immunolabeling of Syk and keratin on paraffin embedded tissue slices, slides were baked overnight at 65°C, and deparaffinized using xylene and a graded series of ethanol. They were then air dried and the tissues were encircled using a PAP pen (Sigma-Aldrich, St. Louis, MO). Slides were rehydrated in phosphate buffered saline, pH 7.4 (PBS) and then submitted to 30 min microwave antigen retrieval using citrate buffer pH 6.0 (Invitrogen cat no. 00-5001) equilibrated in a boiling water bath. The sections were washed for 1 h at room temperature and then endogenous avidin-biotin was blocked (Avidin/Biotin Blocking Kit, Invitrogen cat. No. 00-4303). Non-specific binding of antibodies was blocked for 5 min using PBS containing 10% non-immune goat serum and 0.1% gelatin. Primary antibodies were applied, the sections washed 2× PBS 5 min each, and secondary antibodies were applied in the following steps:

Step 1: 30 min at room temperature: PBS/0.1% gelatin containing 1/100 rabbit monoclonal anti-Syk (clone EP 573Y, Epitomics (1688-1), Burlingame, CA) and 1/100 anti-pan cytokeratin (clone AE1/AE3 mouse monoclonal, Invitrogen cat. No. 18-0132).

Step 2: 15 min at room temperature: PBS/0.1% gelatin containing 1/300 biotin-labeled goat anti-rabbit antibody (Invitrogen cat. No. 50-2352).

Step 3: 15 min at room temperature: PBS/0.1% gelatin containing 1/300 streptavidin AlexaFluor633 (Invitrogen cat. No. S21375, lot 731496), 1/200 rhodamine-labeled goat anti-mouse antibody for 15 min (Sigma), and DAPI (Sigma).

After a final 2× PBS 5 min each wash, the sections were mounted using Fluoro-gel and no. 1.5 coverslips applied (Electron Microscopy Sciences cat. No. 17985-10, Hatfield, PA).

### Confocal Imaging and Syk protein quantification

Fluorescently stained sections were imaged using a Zeiss LSM510/META/NLO microscope equipped with a 10×/0.3 N.A. (initial inspection) or a 25×/0.8 N.A. W lens. For quantitative analysis, images were obtained using the 25× objective with identical imaging parameters for all samples. A series of adjacent images was obtained for all available tissue epithelial areas identified by the pathologist (using H/E adjacent sections) for each stained tissue section. Protein analysis was difficult when comparing tissues using many images from the same slide. Uneven staining across sections occurred and different age of tissue blocks affected staining levels between cases. Therefore, image analysis using keratin to normalize Syk epithelial measurements was carried out to increase the accuracy for reporting protein levels.

For image quantification to determine relative protein levels, average background pixel values were first determined and subtracted for each image to produce background subtracted keratin and Syk images. This was accomplished using a custom Metamorph Offline journal (ver. 7.7.1.0) (MetaMorph® Microscopy Automation & Image Analysis Software, Molecular Devices, LLC, Sunnyvale, CA). Another custom automated journal was used to determine values for keratin, epithelial Syk, and immune cell Syk protein as follows. The average intensity and pixel location of keratin was measured, and then Syk was determined using keratin positive pixels for identification of epithelial cells. Next, the average intensity of immune cells only was determined by filtering for the smaller size of immune cells. For each set of images representing one case, the appropriate threshold level was selected for keratin, epithelial Syk, and immune cell Syk. However, objects less than 70 µm^2^ were excluded from epithelial measurements to minimize the contribution of immune cells to epithelial cell measurements where possible. For selection of immune cells for Syk intensity measurements, areas were excluded that were less than 20 µm^2^ in size, less than 100 average intensity value or greater than 15,000 µm^2^ in area to exclude epithelial cells.

### Statistical Methods

Single factor ANOVA was performed using Microsoft Excel Tools (Data Analysis). Sigma Plot ver. 7.1 was used to plot the data using box plots (means are indicated by red lines within the box and medians are indicated by black lines). The boundary of the box closest to zero indicates the 25th percentile and the boundary of the box farthest from zero indicates the 75th percentile. Whiskers above and below the box indicate the 95th and 5th percentiles. At least three points are required to compute each set of percentiles.

### cBioPortal analysis of TCGA data sets

cBioPortal, a tool developed by the Computations Biology Center at Sloan Kettering, was accessed at http://www.cbioportal.org/public-portal/ [26], [27]. The “Breast Invasive Carcinoma (TCGA, Provisional)” data set was queried (the data set contained 929 total cases at the time of analysis, July, 2013, and 696 cases of IDC were identified with available sequencing and aCGH data). Previously, we determined genes that were up- or down-regulated by *SYK* siRNA knockdown (> = 1.5 fold) in MCF10A cells cultured on collagen I fibrillar matrices [3]. We subsequently curated 51 genes for their relationship to motility and invasion (*ABLIM1, ADAM12, ADAM15, AMOTL2, AP1M2, AXL, CDC42EP4, CHN1, CKAP4, CORO1A, CTNNAL1, CXCL1, CXCL2, ECT2, EGFR, FSCN1, GPER, KIF20A, KIF2C, LAMP1, LAMP2, MARCKSL1, MET, MMP7, MUC1, MYL9, NEBL, PCDH9, RAB11A, RAB11FIP1, RAB20, RAB25, RHOBTB3, RHOD, RND3, RRAS, SMTN, SPRR1A, SPRR1B, SPTBN2, SYK, TIMP1, TIMP2, TNNT1, TPM1, TRAK2, VAMP8, VILL, VIM, WASF1, WASF2*). All of these genes were up-regulated or down-regulated > = 1.5- fold following *SYK* knockdown (Table S1). To this list of SYK-regulated genes, we added *SRC* (c-Src), *CTTN* (cortactin), *CDH1* (E-cadherin), and *TP53* (p53) since the protein products of the first three interact with Syk in a cellular context of motility and invasion; TP53 is part of the interacting network of proteins. The total set of genes is henceforth referred to as the 55 Gene Set. 696 Infiltrating Ductal Carcinoma (IDC) cases were identified using cBioPortal to access de-identified patient information, and cancer subtypes of Her2, basal-like, luminal A, and luminal B were identified as previously determined using PAM50 (known for 675 of the 696 IDC tumor cases) [28]. We obtained 1) Scatter plots for *SYK* copy number, methylation and protein expression and two-gene comparisons; 2) overall survival curves, and “oncoprints” illustrating copy number and mutation data for each patient and 55 genes; 3) data on the % cases altered for each gene; 4) *SYK* copy number, methylation, mRNA, and protein, and 5) a gene interaction network map, including 50 nearest interacting genes with the most alterations, for the 55 gene set using cBioPortal.

## Results

### SYK DNA FISH

**Figure 1:**
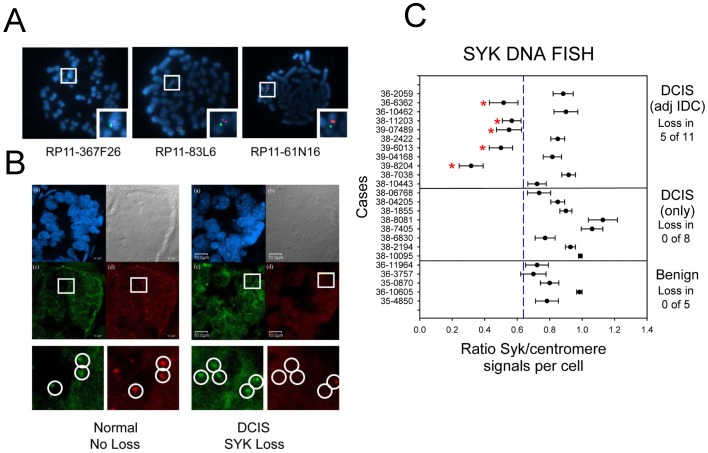
DNA FISH Analysis. **A.** Normal control metaphase spreads were used to validate the *SYK* probe (chromosome 9). The centromeric probe for chromosome 9 is green and the probe for *SYK* is red. Each BAC was labeled with Spectrum Orange™ (a) RP11-367F26, (b) RP11-83L6, (c) RP11-61N16. **B.** Confocal microscopy imaging of FISH slides. The panel at left is an example of normal mammary tissue and the panel at right is of DCIS tissue with *SYK* gene loss. In both, (a) DAPI-stained nuclei, (b) differential interference contrast imaging of section, (c) *SYK* FISH signal (green), (d) chromosome 9 centromere signal (red). **C.** The mean value for the ratio of *SYK*/chromosome 9 centromere signals for each case is illustrated in a bar plot for benign only, DCIS only, and DCIS with adjacent IDC samples. The mean was determined for each case from the ratios obtained individually from 30 cells. Error bars indicate S.E. of the mean. The mean *SYK*/control ratio of the combined cases was 0.92+/−0.048 S.E. for DCIS only tissues and 0.70+/−0.057 S.E. for DCIS adjacent to IDC cases. The mean for benign only tissues was 0.80+/−0.050 S.E.. The ANOVA P value for the three tissues was 0.035.

BAC probes to detect *SYK* DNA were tested using a dual color FISH approach on metaphase spreads of normal human lymphocytes with chromosome 9 centromere control labeled in a different fluorophore (Figure 1A) [22]. Next, the protocol was optimized to detect *SYK* DNA in paraffin embedded sections of breast tissue (Figure 1B). Nineteen cases of breast cancer were selected from a set originally obtained and characterized from the archives at Georgetown University Lombardi Comprehensive Cancer Center [4]. The cases contained DCIS either with or without accompanying evidence of IDC and had the same overall characteristics as the entire set with respect to mRNA Syk expression (Figure 2 in ref. [4]; Figure S1, and Table S2 in this study). De-identified pathology reports and re-examination of sections by an independent pathologist confirmed that 8 cases were DCIS only and 11 cases contained invasive ductal carcinoma (IDC) adjacent to the DCIS (Table S1). For reference, an additional 5 cases were selected that contained benign tissue, but no evidence of DCIS or IDC. Dual FISH analysis for *SYK* was performed on these 24 cases. For the subset of cases used for FISH (Table S2), on average, *SYK* mRNA was reduced comparing benign to DCIS and DCIS to IDC (Figure S1A, original study [4]; 1B, Table 1 subset).

All of the benign epithelium (5 cases of benign with no DCIS or IDC detected) and 8 DCIS tissues from DCIS only cases displayed normal Syk/centromere probe ratios indicating no allelic loss, with a range of 0.7 to 1.1 Syk/centromere probe ratios (Figs. 1B–C). In contrast, 5 out of 11 cases of DCIS in which adjacent IDC was detected displayed a clear loss of *SYK* with a range of 0.3 to 0.6 Syk/centromere probe ratios (Figs. 1B and 1C, asterisks). Thus, for cases in which DCIS was reported to be 100% from the pathology report and subsequent pathologist re-examination, no allelic loss of *SYK* was detected in DCIS tissue. In contrast, in cases in which DCIS was listed at <100% in the pathology report or in which IDC was identified adjacent to DCIS, allelic loss was detected in 5 of 11 cases (46%) (Table S2, Figure 1C, red asterisks). The mean *SYK*/centromere probe ratio of the combined cases was 0.92+/−0.048 S.E. for DCIS only tissues and 0.70+/−0.057 S.E. for DCIS adjacent to IDC cases. The mean for benign only tissues was 0.80+/−0.050 S.E.. Benign was significantly different from DCIS only (P = 0.047) and the DCIS only was significantly different from the DCIS adjacent to IDC (P = 0.0079). These data are consistent with the hypothesis that allelic loss of *SYK* is associated with progression to invasive disease since it occurred more frequently in DCIS with adjacent IDC.

### SYK promoter methylation DCIS tissues

**Figure 2:**
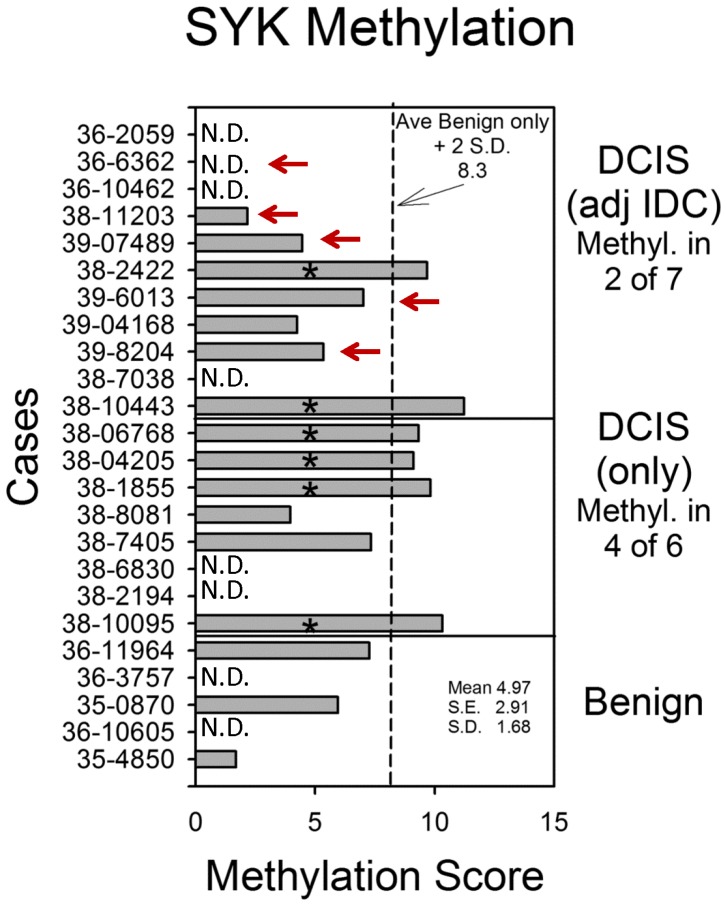
*SYK* promoter methylation. The mean value for promoter methylation at 4 CpG islands was plotted for each case. DCIS cases are arranged by whether adjacent IDC was absent or present, and benign only cases are shown for comparison (mean 4.97+/−1.68 S.D., 2.91 S.E.). The dashed line indicates the mean of the benign cases plus 2 S.D. (8.3). Red arrows indicate cases where FISH identified allelic loss.

Previously, it was reported that CpG islands in the *SYK* promoter were methylated resulting in gene silencing and that methylation of *SYK* was less than 5% in normal or benign tissues, and 47% in node-negative, and 40% in node positive IDC cases [6], [7]. Methylation in DCIS was found to be 45% [7]. We examined the promoter methylation status of *SYK* in DCIS for the present study using bisulphite pyrosequencing using a subset of cases that were available following the FISH study. In 5 benign cases, only three sections were available or yielded to pyrosequencing, only 6 of 8 DCIS only tissues produced results, and only 7 of 11 DCIS tissues from cases with adjacent IDC produced results (Table S2). If a cutoff of 8.3% methylation was set ( = benign only tissue mean +2 S.D., Figure 2, dashed line), 0 of 3 benign cases, 4 of 6 DCIS (only) cases, and 2 of 7 DCIS (with adjacent IDC) cases were considered positive for CpG island methylation (Table S2). Overall, DCIS tissue was positive in 46% of 13 total cases. When means of methylation were analyzed by tissue, benign, DCIS, or IDC, no overall significant differences were observed between them (Kruskal-Wallis, p = 0.48, data not shown). Taken together with the results revealing SYK allelic loss together with promoter methylation, Syk protein levels would likely be impacted.

### Protein Expression of SYK in DCIS tissues

**Figure 3:**
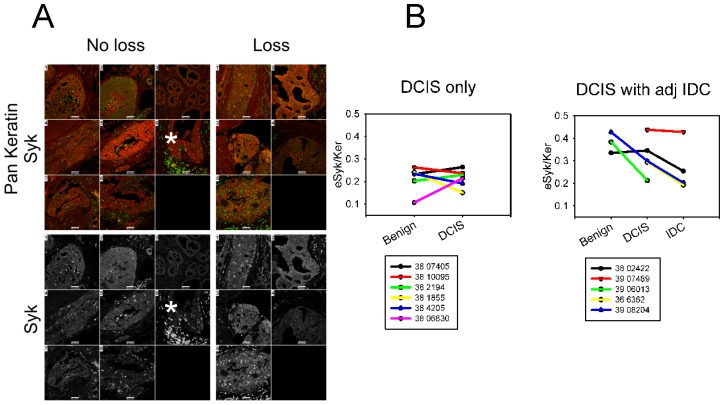
Immunofluorescence staining of DCIS tissues. **A.** Immunofluorescence confocal images of ductal carcinoma in situ (DCIS) tissues from 8 cases with no allelic loss determined by DNA FISH (unless otherwise indicated, DCIS with adjacent IDC): 1) 38 002422 (methylated), 2) 38 06830 (DCIS only, methylation status unknown), 3) 38 10095 (DCIS only, methylated), 4) 38 2194 3 (DCIS only, methylation status unknown), 5) 38 4205 (DCIS only, methylated), 6) 38 7405 A5 (DCIS only, not methylated), 7) 38 8081 8 (DCIS only, not methylated), 8) 39 04168 (not methylated) and 5 cases of DCIS with allelic loss determined by DNA FISH (all DCIS with adjacent IDC: 1) 38 6362, methylation status unknown, 2) 38 11203 (not methylated) 5, 3) 39 06013 (not methylated), 4) 39 07489 (not methylated), 5) 39 08204 (not methylated). Note that anti-Syk staining highlights the presence of a subpopulation of lymphocytes and/or other infiltrating inflammatory cells that is always more intense than tumor epithelium staining (see asterisks). Myoepithelial cells are negative. There was no obvious difference between the intensity of either anti-Syk or anti-pan keratin staining between sections of tissue with or without allelic loss. Scale bars = 50 µm. **B.** The ratios of eSyk/Ker from individual cases were segregated according to DCIS only versus DCIS with adjacent IDC. DCIS only cases are shown on the left (DCIS) and DCIS with adjacent IDC are shown on the right (DCIS with adj IDC). Data was plotted using eSyk/Ker ratios. All available tissues from each case section were used but were not available for every case. Only cases with two or more tissues present were plotted.

In order to examine whether protein loss occurred in parallel with loss of mRNA as previously reported [4], and whether loss was associated with methylation and/or allelic *SYK* gene loss, adjacent tissue sections were double labeled with anti-Syk and anti-pan keratin antibodies and then counterstained with DAPI for immunofluorescence confocal microscopy. Representative quantitative images of DCIS tissues are shown in Figure 3A. Images were analyzed using custom image analysis algorithms (see *Methods*). Determination of the means of average intensities for each case and then pooling all of the means for each tissue, benign, DCIS, and IDC, failed to provide significant data (Figure S2A). Syk-positive immune cells were observed relatively rarely in benign tissue but were particularly abundant infiltrating some DCIS lesions and surrounding IDC (Figure 3A, asterisks). Staining intensity of Syk in immune cells was 6-fold higher than epithelial cells in DCIS, and IDC (Figure S2B).

The ratios of eSyk/Ker for each case were plotted separately to see if individual cases demonstrated Syk loss from benign to DCIS to IDC; the cases were also plotted according to whether they were DCIS only or DCIS with adjacent IDC. If normalized against keratin, epithelial Syk loss is evident in cases of DCIS with adjacent IDC (Figure 3B).

**Figure 4:**
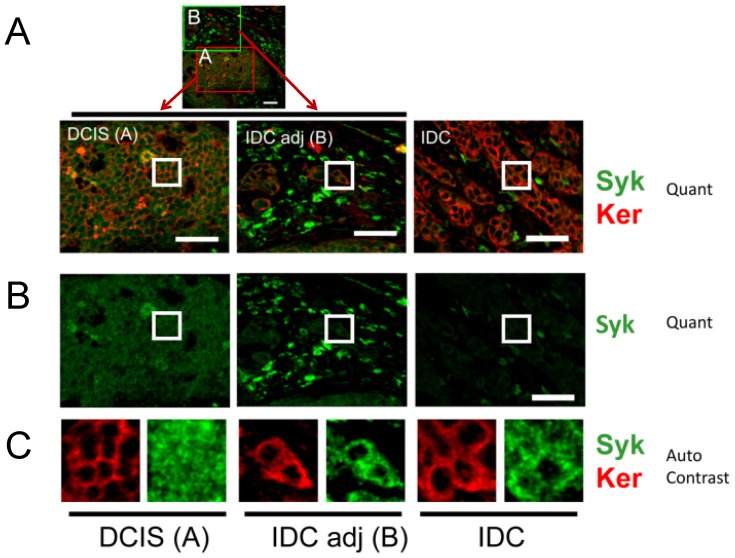
Representative confocal immunofluorescence staining of Syk and keratin in a single case, 36 6362. Two-color images of Syk and keratin staining (Syk,/Ker Quant) are shown for DCIS and IDC tissues within the same confocal image (low magnification view of DCIS with adjacent IDC: “DCIS (A)” and “IDC adj (B)”). IDC from the same slide but a different field of view is shown (“IDC”). The single color, Syk only staining is shown below (Syk, Quant). The background subtracted images were acquired and presented quantitatively for direct comparison (Quant). To qualitatively compare the localization of Syk and keratin, insets are shown on the bottom panels where brightness and contrast were optimized using the auto-contrast function in Metamorph Offline software (Auto Contrast). Keratin is always cytoplasmic, whereas Syk can also be nuclear. The asterisk highlights an area of immune cell infiltration. Scale bars = 50 µm.

Since overall staining intensities varied between cases and over different areas of the slides, we examined single cases (slides) in more detail and focused on a qualitative observation, namely, that Syk occurred both in the cytoplasm and nucleus. Case number 36 6362 contains DCIS with adjacent IDC, lacks methylation of the promoter (value of 7.7 where the cutoff chosen was 8.3), and displays allelic loss (Table S2). Immunostaining of Syk and keratin in a single location and in adjacent locations is illustrated in Figure 4 where epithelial loss of Syk protein is visually apparent, and intense immune cell staining is present (Figure 4A, Syk, Ker, Quant; Figure 4B, Syk, Quant). Insets from the indicated locations in each image were subjected to autocontrast enhancement and are shown in Figure 4C (Syk, Ker, Auto Contrast), revealing the exclusively cytoplasmic staining of keratin (red) versus the cytoplasmic and nuclear staining of Syk (green). Whereas Syk was present in nucleus and cytoplasm in DCIS tissue, it was generally absent in multiple nuclei in IDC tissue (Figure 4C). Analysis of available images for case 36 6362, revealed a significant loss of Syk comparing DCIS and IDC tissues (Wilcoxon rank sum test p-value 0.008). Taken together, the protein staining studies indicate that Syk is lost comparing DCIS and IDC tissues, including nuclear loss.

### Effect of copy number loss on mRNA, methylation, and protein status of SYK invasive breast cancers

To extend the above study on allelic loss of *SYK* in DCIS to invasive breast cancer cases, we took advantage of the large data set publically available on The Cancer Genome Atlas (TCGA, http://cancergenome.nih.gov/) to investigate *SYK* copy number changes. We used cBioPortal tools to access copy number and mutations for *SYK* in this large breast cancer study [26], [28] and to develop a method to discriminate between immune infiltrated invasive breast cancers and cases depleted of immune infiltration so that mRNA and protein values would more accurately reflect epithelial cells (see *Methods*).

**Figure 5:**
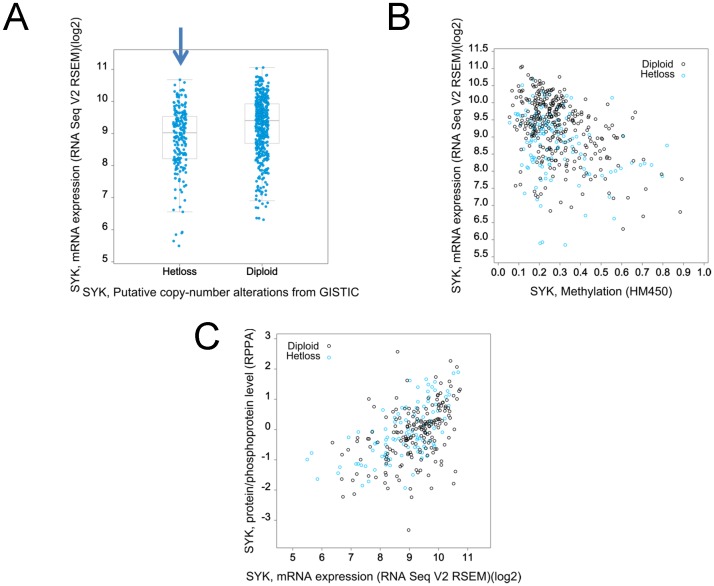
Characterization of the relationship between SYK copy number, mRNA, and protein in immune depleted cases from TCGA Provisional Study. 801 of 1002 breast cancer cases were characterized as immune depleted (see methods) and were used for the following graphs. **A.** Putative *SYK* copy number (x-axis) for Diploid and Hetloss is plotted against *SYK* mRNA in a box plot. The blue arrow indicates the Hetloss group. **B.** *SYK* methylation (x-axis) is plotted against *SYK* mRNA (y-axis) for individual cases of HetLoss or Diploid. Blue circles indicate putative heterozygotic loss and black circles indicate diploid copy number. Dashed line indicates cutoff methylation level. **C.** *SYK* mRNA is plotted against Syk protein/phosphoprotein levels for Diploid and Hetloss cases.

**Figure 6:**
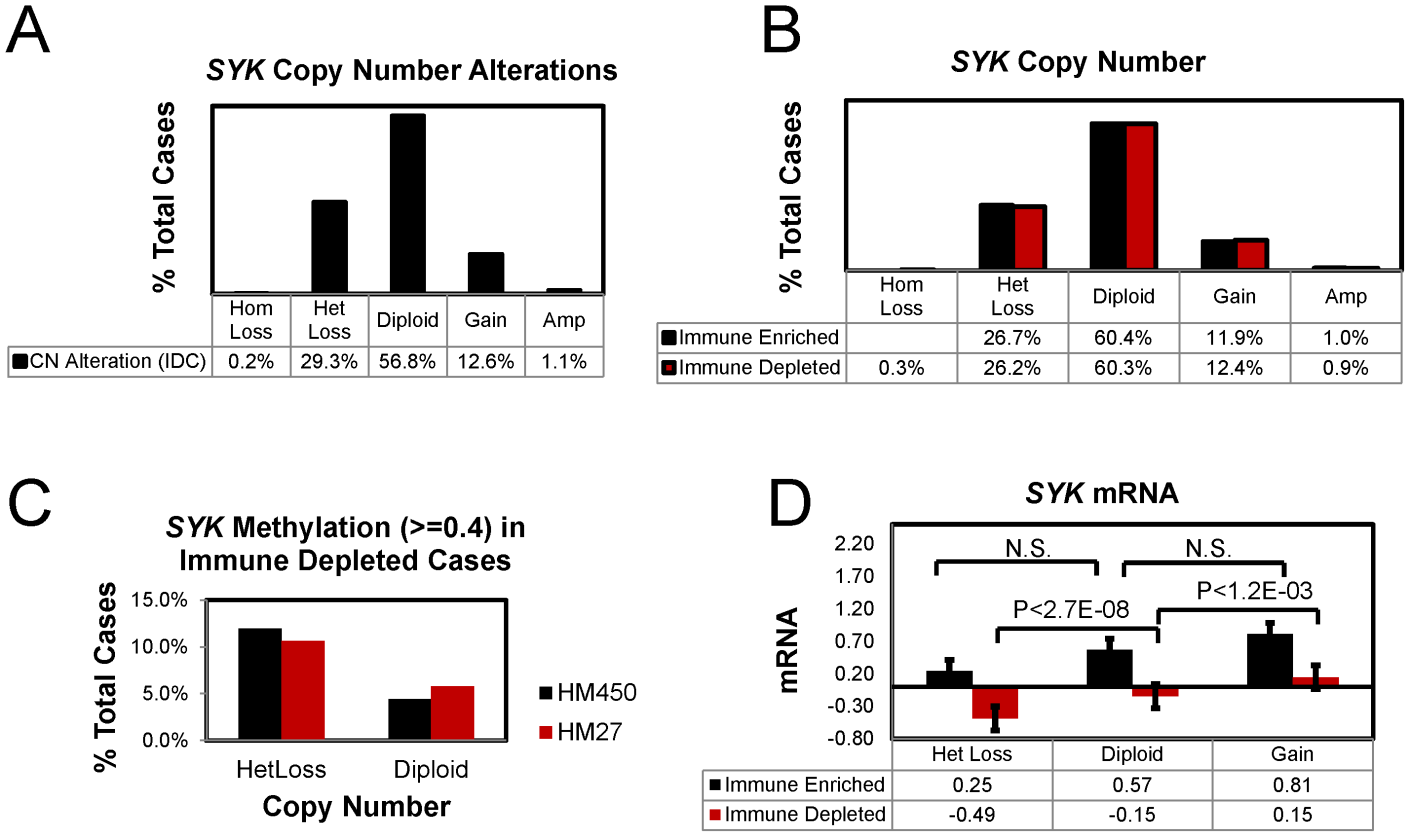
Effect of copy number loss of *SYK* methylation and mRNA expression. **A.** Data for IDC cases were extracted using cBioPortal and the percent of cases for each putative copy number category determined (homozygotic deletion (HomLoss, −2), HetLoss (−1), Diploid, Gain (+1), and amplification (Amp, +2). **B–D.** 1002 invasive breast cancer cases were classified as immune cell depleted (800 cases) or immune cell enriched (202 cases) as described in Methods. **B.** The percent of cases for each putative copy number category was determined. **C.** The percent of cases with methylation > = 0.4 using either the HM450 or the HM27 array data were plotted for immune depleted HetLoss and Diploid cases. **D.** *SYK* mRNA values for each case were extracted and means + S.E. plotted for each category. Only the differences between HetLoss, Diploid, and Gain for immune depleted cases were significant (ANOVA P value 2E-09). P-values for t-tests assuming unequal variances are shown.

Beginning with a total 1002 invasive breast cancer cases from the TCGA, cases where immune cell infiltration was prominent were identified and removed (see *Methods*) for a final subset of 800 immune depleted cases. Using the immune depleted subset or using a subset of 696 IDC only cases, we found that only two mutations in *SYK* were present, one of which would likely have an effect on function, namely A146G [29], [30] (data not shown). The somatic mutation rate for *SYK* was 0.3% and the overall alteration including copy number (homozygotic deletions or amplifications) was 1.6% of IDC cases (696 cases). Putative copy number loss described as heterozygotic loss (HetLoss) occurs in 26.2% of the immune depleted cases (Figure 5A, 6B) and in 29.3% of IDC cases (Figure 6A). As described previously [6], [7], the *SYK* promoter is methylated in invasive breast cancers; and, methylation values were correlated with reduction in *SYK* mRNA levels in immune depleted cases (Figure 5B); similarly protein levels were positively associated with *SYK* mRNA levels (Figure 5C). In immune depleted cases, higher methylation values (> = 0.4) were found in about 11% of HetLoss cases but only in about 5% of diploid cases using either methylation array data (Figure 6C, HM450 or HM27).

In immune depleted, HetLoss cases, a significantly lower level of *SYK* mRNA was observed compared with the Diploid cases; similarly a lower level of *SYK* mRNA was found in Diploid cases compared to Gain cases (Figure 6D). Confirming our identification of a subset of invasive breast cancers where immune infiltration was indicated by alterations in immune specific mRNAs, we found that the mean *SYK* mRNA level was higher in the immune enriched subset of cases compared with immune depleted cases (Figure 6D).

### Poor overall breast cancer survival associated with alterations in genes within the 55 Gene Syk network

#### Influence of SYK on overall patient survival in IDC

To explore the relationship of SYK copy number and mutational status to overall survival in IDC, we asked whether changes in *SYK* status might be associated with a change in overall survival of patients. We queried 696 cases (IDC) from the provisional breast cancer study using mutations and copy number and found that overall survival times estimated in a Kaplan-Meier plot with *SYK* were not significantly different between affected and unaffected cases where affected cases were less than 1% of the total (plot not shown, Logrank test P-value 0.619806).

#### Syk interacting network of 55 genes

**Figure 7:**
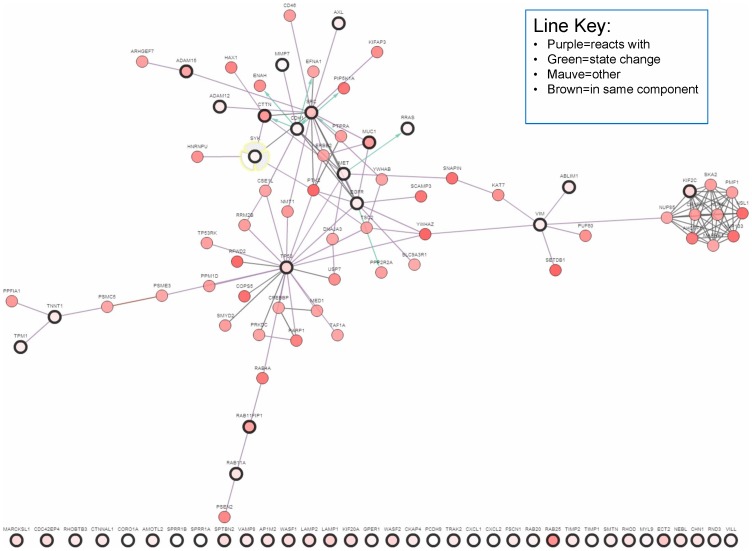
Network interactions amongst the members of the 55 Gene Set. Heavy black circles indicate members of the 55 Gene Set; additional nodes were added based on most related with highest copy number alterations (cBioPortal tools).

We next asked whether *SYK* regulation of gene expression, specifically with regard to its role in suppressing motility, invasion, and metastasis in *in vitro* and *in vivo* models [2], [3], [5], [31]–[33], might be related to the formation of metastases and ultimately patient survival. In a previous study, we had analyzed significant expression changes (up or down regulated > = 1.5 fold) following *SYK* siRNA knockdown in a benign human breast cancer cell line, MCF10A, cultured on a matrix of collagen I [3]. From that set of 708 genes, we next performed a literature search and culled a list of 51 genes involved in key activities required for invasion, including cell motility and activities regulating invasion including membrane trafficking (39 up-regulated and 12 down-regulated genes; Table S1). The addition of *SRC* (c-Src), *CTTN* (cortactin), *TP53* (p53) and *CHD1* (E-cadherin) to the list of 51 genes to form the 55 gene set was based on published data relating these gene products with Syk signaling including direct interactions [4], [31], [34], [35] (Table S1). c-Src tyrosine kinase activity is suppressed by Syk in breast cancer cells [4] and both c-Src and Syk phosphorylate cortactin (*CTTN*) [31], [36]. Syk and cortactin are involved in the development and maintenance of cell-cell contacts and interact with cadherin 1 (*CDH1*) [31], [37], [38]. The relationship between *SYK* and the other 54 genes is shown in an interaction network generated by cBioPortal (Figure 7). 36% of the 55 Gene Set members are linked in a network according to cBioPortal (Figure 7, % altered cases signified by pink intensity, dark-rimmed encircled genes are members of the 55 Gene Set).

#### Functional description of the predictive 55 Gene Set

The 55 genes were annotated using DAVID by investigating Gene Ontology terms (7/19/2013; [39]) to generate a functional annotation table including Biological Function (GOTERM_BP_FAT), Cellular Component (GOTERM_CC_FAT)), and Molecular Function (GOTERM_MF_FAT (Table S3). Prominently, GOTERM terms point to participation in the cytoskeleton, cell adhesion, and association with protein trafficking via intracellular vesicles (Table S3, highlighted terms). Not surprisingly, the actin cytoskeleton and genes that contribute to its regulation are well represented: *ABLIM1*, *CDH1*, *CORO1A*, *CTNNAL1*, *CTTN*, *CXCL1*, *EGFR*, *FSCN1*, *MET*, *MYL9*, *NEB*, *RND3*, *RRAS*, *SMTN*, *SPTBN2*, *TNNT1*, *TPM1*, *VILL*, *VIM*, *WASF1*, and *WASF2*. Many of the GOTERMS are also related to “vesicle-regulating” and “vesicle-associated” proteins (Table S3), for example, *EGFR*, *LAMP1*, *CORO1A*, *LAMP2*, *AP1M2*, *RAB11A*, *RAB11FIP1*, *TIMP1* encode proteins that are vesicle associated proteins and *AP1M2*, *TRAK2*, *TP53*, *RAB11A*, *RAB25*, *RAB11FIP1*, *RAB20*, *KIF20A* genes encode products that participate in protein transport within the cell. Others genes code for cell adhesion genes such as *EGFR*, *CTNNAL1*, *RND3*, *CORO1A*, *PCDH9*, *CDH1*, *ADAM12*, *SRC*, *ADAM15*, and *SYK*. Genes products of *EGFR*, *PCDH9*, *CDH1*, *SRC*, *SYK* regulate cell-cell adhesion; while those of *EGFR*, *MET*, *SRC*, *MYL9*, among other candidate genes, participate in focal adhesion functions.

#### Characteristics of copy number changes in members of the 55 Gene Set

**Figure 8:**
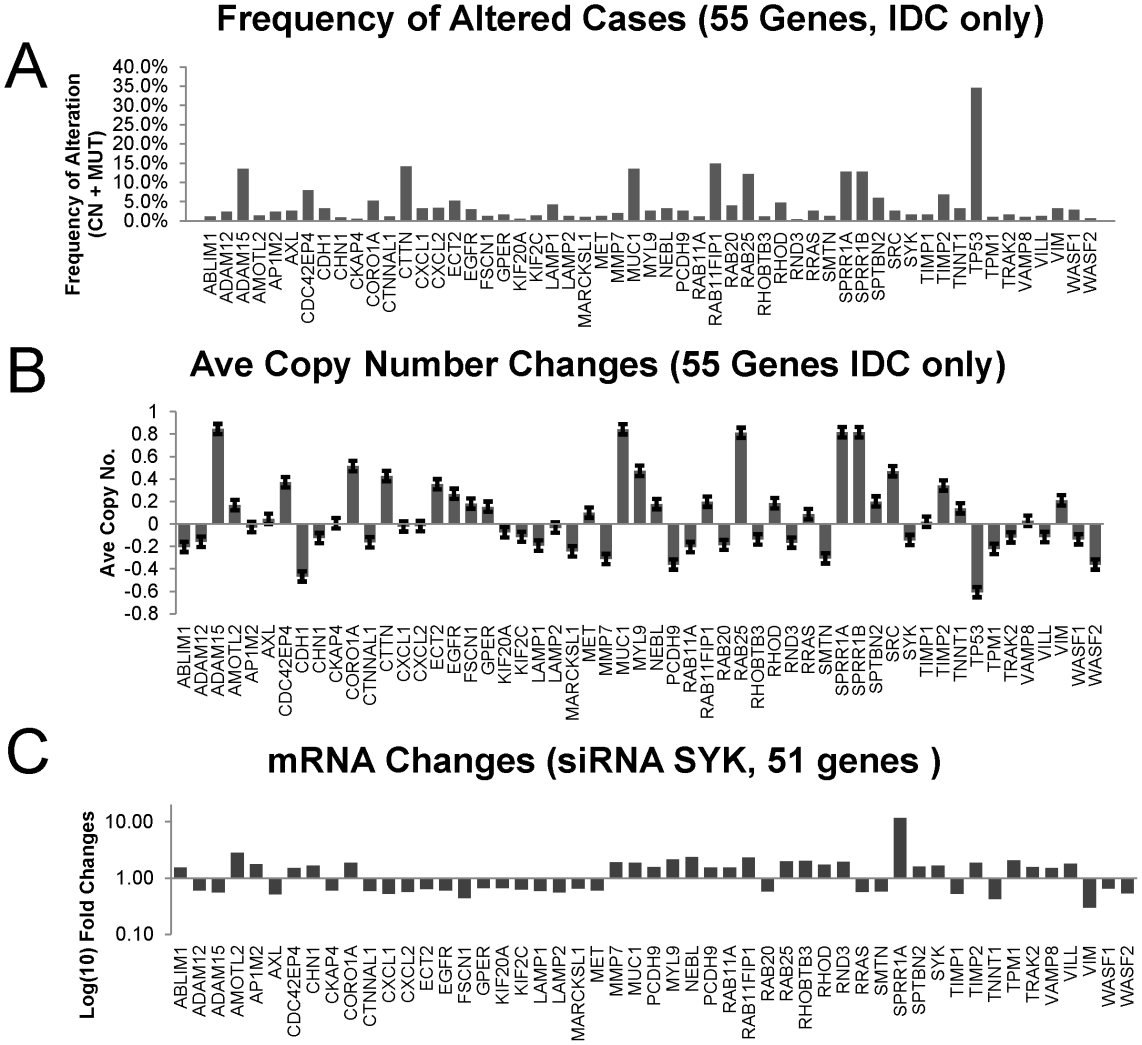
Comparisons of SYK-regulated mRNA changes, average copy numbers, and percent of cases altered. **A.** The % altered IDC cases were plotted for each member of the 55 Gene Set. **B.** The average copy number was plotted for each member of the 55 Gene Set . **C.** Fold changes in mRNAs (log) of genes regulating motility and invasion following SYK siRNA knockdown in ER-negative, MCF10A breast cells [3] were plotted for 51 mRNA species.

To characterize the frequency of the copy number changes in IDC cases and the average copy number of each of the genes from the 55 Gene Set, means were determined and the results were plotted (Figure 8A–B). Of the 9 genes for which the percent of cases altered for the 55 Gene Set was greater than 4.5% (CN, copy number and MUT, mutations) (Figure 8A), all had increased average copy number (Figure 8B); of those, 2 were from genes whose mRNA was down-regulated by *SYK* siRNA (*ADAM15* and *ECT2*) and 7 were from genes whose mRNA was up-regulated by *SYK* siRNA (*SPRR1A*, *RAB11FIP1*, *MUC1*, *RHOD*, *and SPTBN2*). The 8 genes whose copy number frequency alterations (% altered cases) were the greatest were *TP53*, *RAB11FlP1*, *CTTN*, *ADAM15*, *MUC1*, *SPRR1A*, *SPRR1B*, and *RAB25* (Figure 8A). Of these genes, all but one had greater than normal average copy number, TP53 being the exception (Figure 8B). The *SYK* mRNA changes in motility and invasion genes resulting from siRNA treatment of MCF10A cells were plotted for comparison (Figure 8C).

An “oncoprint” illustrates the genomic copy number and mutational changes for each patient and for each of the 55 genes (Figure S3A). This graphic illustration of genes versus patients with amplifications in red, mutations in green, and deletions in blue reveals the pattern of one or more alterations per affected patient (Figure S3A). A curious feature instantly observed upon inspection of the Oncoprint generated by the cBioPortal analysis is that 5 of the amplified genes amongst the 8 with highest frequency of altered cases were co-expressed in the same patient cases (Figure S3A, blue boxed region, and Figure S3B, two gene comparisons). It transpires that these 5 genes are all found on chromosome 1 (SPRR1A: 1q21-q22; SPRR1B: 1q21-q22; ADAM15: 1q21.3; MUC1: 1q21; RAB25: 1q22). Three of these genes, *ADAM15*, *MUC1*, and *RAB25* are part of an interactive network of genes (Figure S3C).

When cases were stratified according to *SYK* copy number (−1 and −2, 0, +1 and +2) and then were used to compare differences in the average copy number for each member of the 55 Gene Set, both *SYK* and *CTNNAL1* (alpha-catulin) copy number had parallel changes suggesting that these two genes are linked (Figure S4A). A two gene comparison using cBioPortal for copy number of *SYK* and *CTNNAL1* resulted in a positive correlation in IDC patients for these two genes (Figure S4B). Both are found on chromosome 9 (SYK: 9q22; CTNNAL1 (catenin (cadherin-associated protein), alpha-like 1, alpha-catulin: 9q31.2).

We conclude that co-amplification and co-loss of genes within adjoining stretches of DNA contribute to genetic disruption of the 55 genes.

#### Identification of breast cancer cases without immune cell infiltration

Syk protein localizes in the luminal ductal epithelium of the breast ([3], [4], therefore we narrowed the cases to include only IDC (infiltrating ductal carcinoma), a subset of 696 cases of the total. Using a set of 9 genes as immune cell markers (*IL2RA*, *CMTM2*, *FCGR2A*, *FCER1A*, *FCER1G*, *WAS*, *CD3E*, *CD22*, and *CD19*) as described in the *Methods*, we queried the IDC cases for alterations based on mRNA and estimate that tissue from 131 cases were contaminated with immune cell contributions (and the calculated number of altered cases was 90.2% of the total 131). The remaining IDC 565 cases were “immune cell depleted” since the 9 genes were altered in only a calculated 4.3% of those cases. Therefore, about 19% of the IDC cases contained significant immune cell infiltration. When this analysis was also performed on the total collection of invasive breast cancers, we found a similar frequency; 20.2% cases including all breast cancer subtypes were immune enriched leaving 800 cases classified as immune depleted.

#### Predictive power of the 55 Gene Set

**Figure 9:**
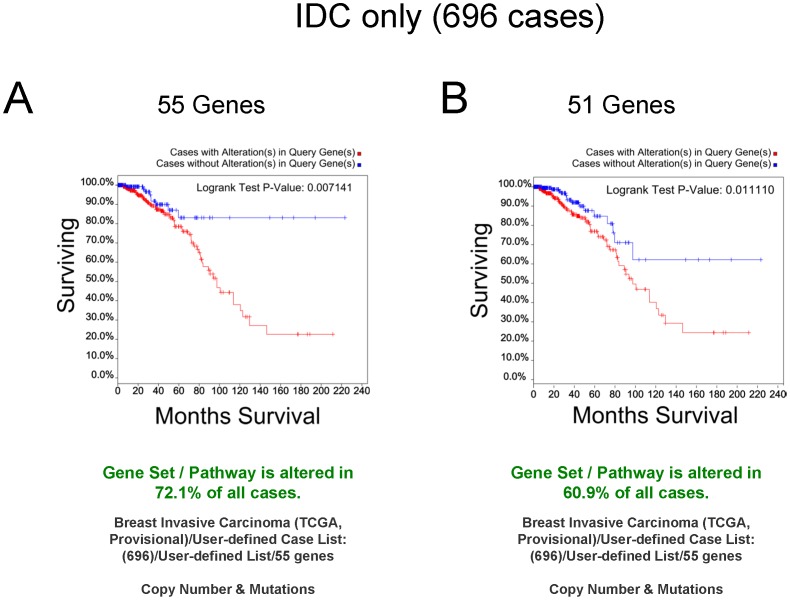
Kaplan-Meier curves of queries of IDC only cases. **A.** Survival curves for 696 cases (IDC cases only) from the TCGA Provisional Breast Cancer Study were generated using cBioPortal querying with the 55 Gene Set. 72.1% of all cases were altered (copy number and mutations). The Logrank test p-value was 0.007141. **B.** The cBioPortal query was repeated using only the 51 genes from the *SYK* microarray study [3]. 60.9% of all cases were altered (copy number and mutations). The Logrank test p-value was 0.011110.

We first analyzed IDC only cases to determine overall survival using copy number and mutation alterations based on the 55 Gene Set. In this analysis, 72.1% of the cases were altered and, impressively, using a Kaplan-Meier analysis, the Logrank test p-value was significant (0.007141) with the curves differentiating between altered cases whose % surviving cases plateaued at ∼20% and unaltered cases at ∼80% (Figure 9A). To ask whether the presence of immune cells might be masking the results in terms of the overall patient survival using the 55 Gene Set, the subset of 565 cases of IDC, depleted of cases with immune contamination, were queried again in the same way. The results were similar curves on the Kaplan-Meier plot with increased significance compared with a query of the entire IDC case set (Immune depleted IDC: Kaplan-Meier plot not shown, Logrank test p-value of 0.003113) and the curve depicting no alterations plateauing at 85% overall survival. Thus, focusing on the copy number and mutational alterations in the 55 Gene Set allows overall survival prediction for the entire set of cases whether or not immune cell infiltration is a prominent component of the extracted tissues. This result indicates a significant decline in survival in affected cases when copy number and mutations (but not mRNA or protein) were considered for these genes. In order to determine the contribution of the four genes whose expression levels were not significantly different according to our previous microarray study, we performed the analysis on just the 51 genes whose mRNAs were significantly regulated by *SYK* knockdown (Figure 9B). The Logrank test p-value was also significant (0.011110) but the survival plateau for unaffected cases was reduced to 60%. We conclude that the 55 Gene Set has higher predictive value than the set of 51 genes alone.

**Figure 10:**
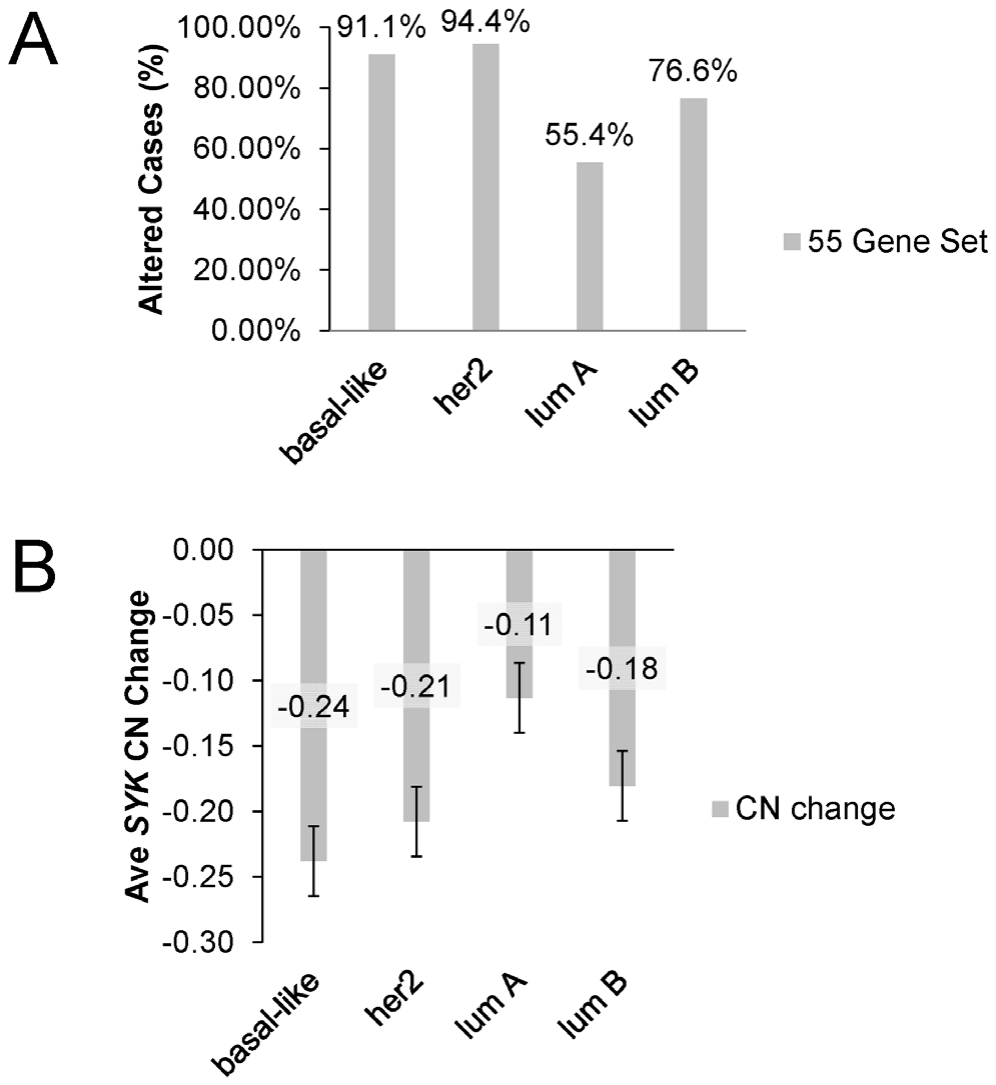
Stratification of cases by breast cancer subtype and by *SYK* copy number and frequency of alteration. **A.** The % of altered IDC cases was determined using the 55 Gene Set for cases identified as basal-like, her2, luminal A or luminal B using the PAM50 gene set [28]. **B.** The average SYK copy number change (CN) was determined for cases identified as in **A.**

In a previous global characterization of the majority of these breast cancer cases from the TCGA, breast cancer subtypes were characterized using PAM50 ([28], and references therein). We performed queries using the 55 Gene Set to compare basal-like, Her2, Luminal A, and Luminal B tumor types. The Gene Set was altered in 91.1% and 94.4% of basal-like and Her2 cases, respectively, whereas 55.4% and 76.6 percent of luminal A and luminal B cases, respectively, were altered (Figure 10A). Thus, these motility- and invasion-related genes are already altered in nearly all cases of these tumor types with known poorer outcome. A similar trend was observed for the average *SYK* copy number change in these cancer subtypes (Figure 10B). Average basal-like and Her2 tumor copy number changes were −0.24 and −0.21, respectively, but were −0.11 and −0.18 for luminal A and luminal B tumors, respectively (Figure 10B). In summary, change in the average *SYK* copy number for each subtype reflects the change in percent of cases with genomic alterations in members of the 55 gene set. We conclude that *SYK* copy number changes reflect the overall genomic instability in the tumors.

**Figure 11:**
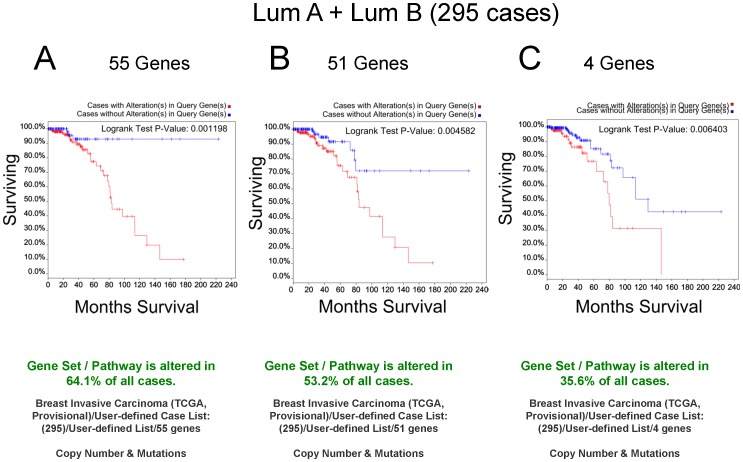
Kaplan-Meier curves of queries of Luminal A + Luminal B cases. **A**. A query of 295 cases of luminal A and luminal B (as determined in [28] using the PAM50 gene set) from the Provisional Breast Cancer Study was performed using the 55 Gene Set. 64.1% of all cases were altered (copy number and mutations). The Logrank test p-value was 0.001196. **B**. The cBioPortal query was repeated using the 51 genes from the *SYK* microarray study [3]. 53.2% of all cases were altered (copy number and mutations). The Logrank test p-value was 0.004582. **C**. The cBioPortal query was repeated using 4 genes (*CDH1*, *CTTN*, *SRC*, and *TP53*). 35.6% of all cases were altered (copy number and mutations). The Logrank test p-value was 0.006403.

We next performed queries to determine overall survival in Luminal A + Luminal B subtypes (295) using 55 Gene Set. The Kaplan-Meier Logrank test p value was 0.001198, and % surviving cases plateaued at ∼10% and unaltered cases at ∼90% (Figure 11A). There was no significant predictive power of the 55 Gene Set for the Her2 or basal-like subtypes (not shown). To parse out the effects of the additional 4 genes (CDH1, CTTN, SRC, and TP53), Kaplan-Meier curves were generated revealing significant Logrank p-values of 0.004582 and 0.006403 for the 51 Gene Set and 4 Gene Set, respectively, with associated reductions in the survival plateaus (Figure 11B–C). Thus, the 55 Gene Set significantly predicts overall survival in luminal breast cancer subtypes but not in basal-like or Her2 cases where the majority of cases already have alterations in these genes.

## Discussion

### Significance of results: allelic loss of SYK

For the first time, allelic loss of *SYK* was detected in DCIS breast tissues using a subset of the tissues in which progressive loss of *SYK* mRNA from benign to DCIS to IDC tissues was reported [4]. Remarkably, in this small study, 46% of DCIS cases displaying adjacent IDC, but none of the DCIS only cases, were found to have allelic gene loss of *SYK*. Thus, in this preliminary study of DCIS, there appears to be a strong association of *SYK* allelic loss in DCIS tissues with adjacent invasive breast cancer. Using a large, publically available data set, we confirm *SYK* loss at the genomic level in IDC and found that about 30% of IDC cases displayed heterozygotic *SYK* loss. The frequency of *SYK* loss in DCIS and IDC tissues suggests that it could play a role in enhancing metastatic progression in a significant number of cases.

### SYK mRNA loss in cases of reduced copy number: a method to examine cases depleted of immune cell infiltration

To determine the consequence of *SYK* copy number loss on Syk expression and thus the potential of Syk to regulate its downstream targets, it was critical to isolate Syk regulated events occurring in epithelial cells from those operative in immune cells. A key source of artifact in studies of Syk arises when tissue samples are taken and used to analyze genomic DNA, mRNA, and protein because of immune cell infiltration. We found that protein levels of Syk were 6-fold higher in immune cells compared with epithelial cells. In this study of TCGA data, we estimated that about 19% of the IDC cases had significant immune cell infiltration that contributed to increased immune cell mRNA and proteins isolated from tissue extracts. Our success at isolating immune enriched versus immune depleted cases was confirmed by observation of elevated levels of *SYK* mRNA in the immune enriched compared with the immune depleted cases. Analyzing immune depleted cases, we were therefore able to ascertain that a larger proportion of HetLoss *SYK* cases had *SYK* promoter methylation compared to diploid *SYK* cases. Furthermore, and importantly, the mean *SYK* mRNA level was lower in HetLoss compared with Diploid cases. We therefore conclude that *SYK* copy number loss and promoter methylation leads to reduction in Syk expression. This evidence from human pathological tissues agrees with the results generated from *in vivo* mouse studies and *in vitro* human tissue culture studies that *SYK* allelic loss results in reduced *SYK* expression [3]. We propose that *SYK* loss in human breast tumors *in situ* may also result in loss of Syk suppression of motility and invasion to support metastatic progression in breast cancer patients via its action upon proteins regulating motility and invasion.

### Impact of SYK-regulated motility and invasion gene network on breast cancer survival

Independent of the effect of Syk function on the network of Syk-interacting proteins, increased genomic instability contributing to changes in copy number and mutations of these 55 genes would also have an outcome contributing to metastatic progression. This hypothesis is supported by the observation of a significantly lower overall survival in IDC cases, and specifically in Luminal A plus Luminal B subtypes in cases where members of the 55 Gene Set are altered. Considering the difficulty of performing mRNA and protein studies on breast cancer tissue homogenates, it was fortunate that these genes could be used to assess overall survival based solely on copy number and mutations. Genomic changes would be less sensitive to the presence of immune cell DNA in the tissue lysates compared with the more abundant mRNA or protein species in the cytoplasm. We conclude that Syk-regulated genes represent an epithelial biological network for restraint of motility and invasion that contribute to suppressing metastasis; in turn, loss of this restraint due to genomic instability or loss of Syk expression decreases overall survival of breast cancer patients. Evaluation of the 55 Gene Set for prognostic information or predictive power for treatment outcome should be considered in the future.

Our identification of a 55 Gene Set that potentially represents a new prognostic tool is based on *SYK* and the identification of the important biological function of Syk to regulate epithelial cellular invasion. This is in contrast to prognostic sets such as the PAM50 and the MammoPrint which were selected based on different strategies. Interestingly, there was no overlap in genes of the 55 Gene Set with 164 of the identified genes in Table S2 reported by Van't Veer et al, and later referred to as MammoPrint [40]. There was some overlap with the PAM50 gene set, namely *AP1M2*, *CDH1*, *CORO1A*, *ECT2*, *LAMP2*, *MMP7*, *MUC1*, *NEBL*, *RAB11A*, *SYK*, *TIMP2*, *TNNT1*, and *VIM* underscoring the importance of these epithelial invasion/motility genes in patient outcome.

### Impact of SYK-regulated motility and invasion gene network on breast cancer survival in cancer subtypes and relationship to estrogen receptor status

The 55 Gene Set appears to be most potent in predicting survival in luminal IDC subtypes (luminal A and luminal B case assignments determined by PAM50 [28]); alterations in the members of the 55 Gene Set resulted in reduced overall survival. However, the 55 Gene Set analyses found that the majority of basal-like and Her2 cases were already altered so that a comparison of altered versus non-altered cases was not useful. A main feature of luminal subtype breast cancers is that they are, for the most part, estrogen receptor (ER) positive. In this study, 478/675 (71%) of IDC cases and 284/295 (96%) of luminal A and luminal B cases were ER+. If IDC cases were stratified by ER+ versus ER− and queried using the 55 Gene Set, only the ER+ cases were significantly different as estimated by Kaplan-Meier curves (Logrank test p-value 0.003212 for ER+, n = 478 cases and 0.700637 for ER−, n = 197 cases). In a study of a series of breast cancer cell lines that were ER+ versus ER−, Syk expression was mostly positive in ER+ and negative in ER− cell lines [2]. Cell lines lacking Syk were invasive in a 3D test of invasion, whereas those with Syk expression were non-invasive [2]. Thus, basal-like cell lines such as MDA-MB-231 are ER− and highly invasive and already are Syk depleted [2]. However, the correlation between ER+ and SYK+ cell lines is not perfect. MCF10A cells used for the SYK knockdown experiments [3] are Syk+ but ER− and so represent one of the exceptions. Even so, the progression series of MCF10A cell lines shows a loss of Syk in the DCIS.COM line compared with MCF10A; and Syk knockdown in either cell line results in increased proliferation and invasion characteristics [3]. Thus, although the functional relationship between Syk and ER is not clear, Syk regulation of motility and invasion activity is independent of ER at least in the case of MCF10A. Interestingly, ESR1 protein and phosphoS118 ESR1 (ERα or estrogen receptor 1) are decreased in cases where alterations in the 55 Gene Set are present (Table S4). That these tumors are more aggressive is also suggested by recent data using anti-phospho S118 ESR1 to identify cases where the ERα pathway is active; mutation of this site leads to more aggressive cell behavior in MCF7 breast cancer cells ([41] and references therein).

### Biological functions of the 55 Gene Set Members

Members of the 55 Gene Set had been chosen for their roles in events driving motility and invasion, and thus contributing to metastasis. Many members of these Syk-regulated genes from the 55 Gene Set have already been reported in the context of Cancer and Breast Cancer studies (Figure S5). Src, Syk, EGFR, and MET regulate invasion-promoting activities including invadopodia-mediated matrix degradation via MT1-MMP (MMP14) and cell-cell and cell-extracellular matrix adhesion (including integrin heterodimers and cadherins) to determine the invasive phenotype [42]–[52]. Five of these *SYK*-regulated genes encode kinases that are proto-oncogenes (*EGFR, MET, AXL, ECT2, and SRC*) two of which (*EGFR* and *SRC*) have previously been linked to Syk in epithelial cells [4], [53]. Specifically, we found earlier that the cell surface expression of invadopodia genes of an integrin heterodimer, alpha6beta1 (CD49f, ITGA6/ITGB1), adhesion receptor CD44, and matrix-degrading protease, MT1-MMP (MMP14), are directly regulated by SYK; SYK knockdown results in their increased expression at the cell surface coupled with the formation of invadopodia and consequent cell invasion [3]. Src and Syk both interact with integrins and interact with each other to regulate tumor cell invasion [4], [54], [55]; Src-substrates are well known contributors to the invasive function of invadopodia [2], [3], [5], [42], [56], [57]. ADAM15, up-regulated following SYK knockdown in MCF10A cells, was identified in this study as frequently altered in copy number. ADAM15 functions in invadopodia by binding TKS5, a Src substrate; variants contribute to mammary cell carcinoma, and increased copy number was found to lead to formation of multiple variants [57]–[59]. *RAB25* and *RAB11*, closely related GTPases, and the RAB-binding protein *RAB11FIP1* regulate endosomal traffic, contribute to integrin recycling to the cell surface, to Src positioning in the cell, and to many other endosomal trafficking events related to invasion. Our data agrees and supports other reports of the important role of membrane trafficking for adhesion, motility, and invasion specifically involving RAS family GTPases [60]–[76].

Another major theme is the enrichment of genes whose products are associated with or regulate the actin-cytoskeleton. *CTTN* (cortactin) is a gene that encodes an-actin binding protein that is also a prominent Src tyrosine kinase substrate. The c-Src-cortactin interaction impacts invasion including its central role in invadopodia formation and function and vesicle trafficking, specifically delivery of matrix-degrading proteases to the cell surface [77]–[80]. *CTTN* gene amplification in cancer has previously been reported [81], [82].

Another subset of SYK-regulated genes in the 55 Gene Set is important for cell invasion mechanisms including matrix degradation and adhesion. These include *MMP7, ADAM12, and ADAM15* metalloproteinases, the latter two of which contribute to the activity of invadopodia and possess disintegrin adhesion domains. Also included are the tissue inhibitors of metalloproteinases *TIMP2* and *TIMP1*; they are metalloproteinase inhibitors that regulate the activity of extracellular matrix degrading MMP-9, MMP-2, and MT1-MMP (MMP14) [83], [84].

Gain of 1q in cancer, specifically in breast cancer has previously been described [28] but is impressively evidenced in this study by frequent co-amplification of the SYK-regulated *SPRR1A*, *SPRR1B*, *MUC1*, *RAB25*, and *ADAM15*, genes located within chromosome 1q. We have also made the novel observation of *SYK* and *CTNNAL1* (alpha-catulin, located on chromosome 9q) co-loss in this study. Alpha-catulin is an E-cadherin-regulating cell-cell adhesion protein that has been recently implicated in melanoma invasion and metastasis [85].

### Conclusions

In conclusion, we have demonstrated that *SYK* allelic loss occurs both in DCIS tissues as well as IDC tissues. Immune cell Syk protein is 6-fold greater than epithelial Syk. This complicates analysis of tissue homogenates from the TCGA. However, we demonstrated that heterozygotic *SYK* is associated with reduction in *SYK* mRNA compared with diploid cases in breast cancer cases without immune filtration. We accomplished this using our new method to identify cases where immune cell infiltration is present. Finally, we identified a 55 Gene Set, the Syk interacting network of invasion-related genes. This gene set powerfully predicted poorer patient outcome when copy number and mutational alterations of one or more of these genes occurred. In summary, we propose two mechanisms whereby *SYK* loss together with mutations and copy number changes in the 55 Gene Set results in poorer overall survival of patients.

## Supporting Information

Figure S1 **Syk mRNA** ***In Situ*** **hybridization.** **A.** *In situ* data from our original study of *SYK* mRNA [4] is graphed as mean values for benign only, all DCIS (DCIS), and IDC tissues. ANOVA P-value = 2.91E-11 for the three tissues. Both DCIS and IDC were significantly different from Benign tissues (3.95E-06 and 7.38E-10, respectively) and DCIS was significantly different from IDC (0.013). Scores for benign (mean 2.67+/−0.11 S.E.), DCIS (mean 1.84+/−0.13 S.E.), and IDC (mean 1.29+/−0.17 S.E.) tissues are shown. Box plots were generated using Sigma Plot: red lines indicate the mean, black lines the median, and a 95%/5% range is indicated by whiskers. Boxes represent the 75%/25% range. **B.** The *in situ* data for the subset of cases shown in Table S2 were analyzed as in **A**. and graphed as means of Benign, DCIS and IDC tissues. The results are representative of the data and analysis obtained for the entire set of cases originally published [4]. ANOVA P-value = 0.00027 for the three tissues. Both DCIS and IDC were significantly different from Benign tissues (0.0048 and 0.0072, respectively). DCIS and IDC were not significantly different (0.077184). Scores for benign (mean 2.77+/−0.17 S.E.), DCIS (mean 1.84+/−0.25 S.E.), and IDC (mean 0.75+/−0.48 S.E.) tissues are shown. Box plots were generated using Sigma Plot: red lines indicate the mean, black lines the median, and a 95%/5% range is indicated by whiskers. Boxes represent the 75%/25% range. All t-tests were two-tailed.
(PDF)

Figure S2 **Quantitative immunofluorescence determination of protein.** **A.** Mean intensity values were obtained as described in the *Methods* for each case by tissue type: Benign, DCIS, and IDC. Those means were averaged; the results for Syk in epithelial cells (eSyk), immune cells (eSyk) and keratin (Ker) are shown in the same graph to illustrate the relative staining for Syk in epithelial versus immune cells and as compared with keratin. Box plots were generated using Sigma Plot: red lines indicate the mean, black lines the median, and a 95%/5% range is indicated by whiskers. Boxes represent the 75%/25% range. **B**. The iSyk/eSyk protein ratios for benign, DCIS and IDC cases were graphed as a box plot. The mean and standard error (S.E.) are shown in the table below.
(PDF)

Figure S3 **Co-alterations in 5 genes located on chromosome 1.** **A.** Oncoprint generated using cBioPortal of genomic copy number and mutational changes in the members of the 55 Gene Set. **B.** Two gene comparisons of *ADAM15* (x-axis) against *RAB25*, *SPRR1A*, and *MUC1*, respectively. C. Network of interactions of *ADAM15*, *RAB25*, *SPRR1A*, *SPRR1B*, and *MUC1* including the 50 most mutated genes within the network.
(PDF)

Figure S4 **Average copy number of members of the 55 Gene Set in IDC cases stratified by** ***SYK*** **copy number.** **A.** Plot of average copy number (y-axis). **B.** Two gene plot of copy number for *SYK* and *CTNNAL1* both of which are located on chromosome 9.
(PDF)

Figure S5 **PubMed citations for members of the 55 Gene Set AND “Cancer” or members AND “Breast Cancer”.** The number of citations for each gene was determined using PubMed for citations including both the gene symbol and the term “Cancer” (black bars) or “Breast Cancer” (red bars) and the results graphed. The results were sorted by Breast Cancer results with the most frequently cited breast cancer gene being *EGFR*. About half of the genes had been cited for breast cancer at least 5 times. 27 of the Genes were cited less than 5 times. One gene, *SPTBN2* was cited 0 times for cancer and breast cancer.
(PDF)

Table S1 **Summary of motility/invasion gene and mRNA data.** Microarray data on significantly regulated mRNAs following SYK knockdown in MCF10A cells were obtained in a previous study [3]. Data on number of cases altered, % of cases altered and the average copy number gain or loss were determined from TCGA data as described in the *Methods* and *Results*. For number and % cases altered, copy number and mutational changes were evaluated using the cBioPortal software to determine and export this information for the 55 Gene Set. The table is organized by greatest mRNA fold changes to least with losses as numbers >1.5 fold change and gains as <0.67 fold change (VIM the greatest fold increase and SPRR1A the greatest fold decrease in mRNA expression after SYK knockdown).
(PDF)

Table S2 **Summary of FISH,** ***in situ*** **, and methylation data for SYK in tumor epithelium.** Cases were separated by whether they contained DCIS only, DCIS with IDC, or benign only tissues. *SYK* allelic loss, mRNA *in situ*, methylation, and Syk protein values where available are shown. Scores reflect the tissue types present in each case (slide) and available for analyses. For FISH results, the % DCIS was obtained from available pathology reports from the Histopathology and Tissue Shared Resource (second column). The number of FISH signals from the Syk and chromosome 9 centromere probes was determined microscopically by a licensed cytopathologist and their average ratio calculated which is shown in column 3 (J.B.). A cutoff point for “normal” versus “allelic loss” in DCIS tissues from each case was determined from Figure 1, as indicated. *In situ* hybridization results for *SYK* mRNA were obtained previously (Normal, Hyperplasia, DCIS, and Invasive in situ, columns 4–6 [4]. In the next to last set of data (columns 7–9), methylation results are shown. Raw Syk protein values were obtained from image analysis and are shown in the last columns (columns 10–12).
(PDF)

Table S3 **Functional Annotation Table for the 55 Gene Set Members.** A functional gene annotation table was generated using DAVID (http://david.abcc.ncifcrf.gov/) to illustrate the groups of genes involved in motility- and invasion-related activities. Some of the important activities are highlighted.
(PDF)

Table S4 **Protein/phosphoprotein results from immune depleted all types or IDC only cases when queried using cBioPortal for the 55 Gene Set.** Immune depleted invasive breast cancer cases (800 cases) or immune depleted IDC only cases (565 cases) were queried using the 55 Gene Set for mutation and copy number changes. The significant protein and phosphoprotein changes comparing the altered versus nonaltered cases were extracted using cBioPortal.
(PDF)
