# Supplementary figures and images for: SYK Allelic Loss and the Role of Syk-Regulated Genes in Breast Cancer Survival
Source: PLoS One. 2014 Feb 11;9(2):e87610. doi: 10.1371/journal.pone.0087610 (PMC3921124; doi:10.1371/journal.pone.0087610)

Figure S 1

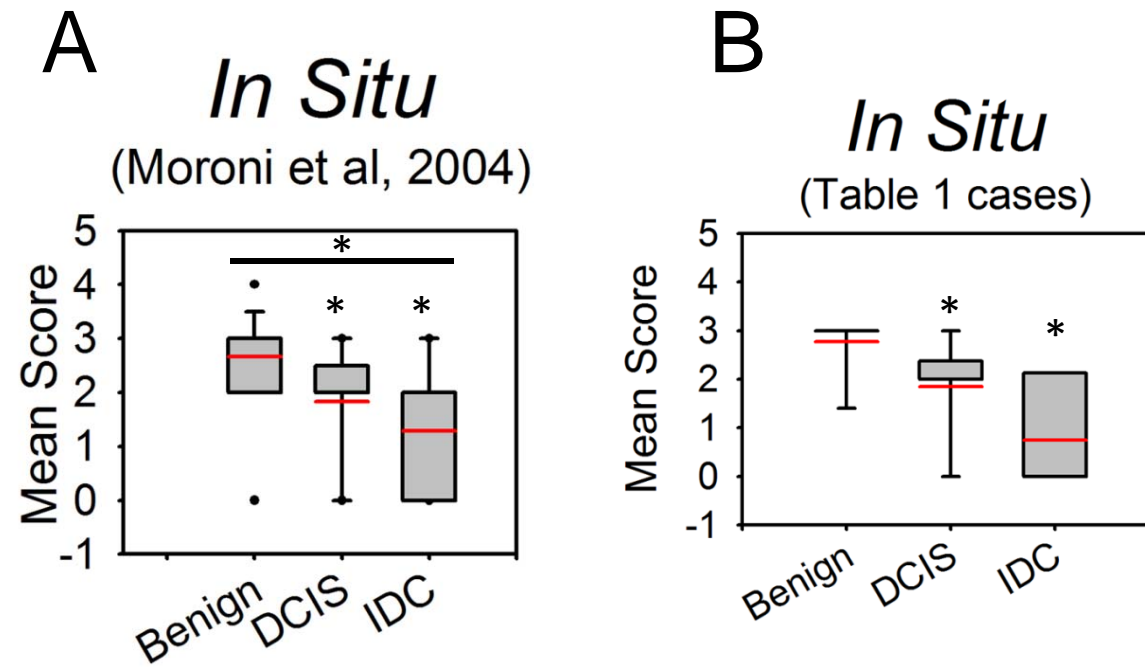

Supplement: Figure S1 — Syk mRNA In Situ hybridization. A. In situ data from our original study of SYK mRNA [4] is graphed as mean values for benign only, all DCIS (DCIS), and IDC tissues. ANOVA P-value = 2.91E-11 for the three tissues. Both DCIS and IDC were significantly different from Benign tissues (3.95E-06 and 7.38E-10, respectively) and DCIS was significantly different from IDC (0.013). Scores for benign (mean 2.67+/−0.11 S.E.), DCIS (mean 1.84+/−0.13 S.E.), and IDC (mean 1.29+/−0.17 S.E.) tissues are shown. Box plots were generated using Sigma Plot: red lines indicate the mean, black lines the median, and a 95%/5% range is indicated by whiskers. Boxes represent the 75%/25% range. B. The in situ data for the subset of cases shown in Table S2 were analyzed as in A. and graphed as means of Benign, DCIS and IDC tissues. The results are representative of the data and analysis obtained for the entire set of cases originally published [4]. ANOVA P-value = 0.00027 for the three tissues. Both DCIS and IDC were significantly different from Benign tissues (0.0048 and 0.0072, respectively). DCIS and IDC were not significantly different (0.077184). Scores for benign (mean 2.77+/−0.17 S.E.), DCIS (mean 1.84+/−0.25 S.E.), and IDC (mean 0.75+/−0.48 S.E.) tissues are shown. Box plots were generated using Sigma Plot: red lines indicate the mean, black lines the median, and a 95%/5% range is indicated by whiskers. Boxes represent the 75%/25% range. All t-tests were two-tailed. (PDF) [file pone.0087610.s001.pdf]

Figure S 2

A

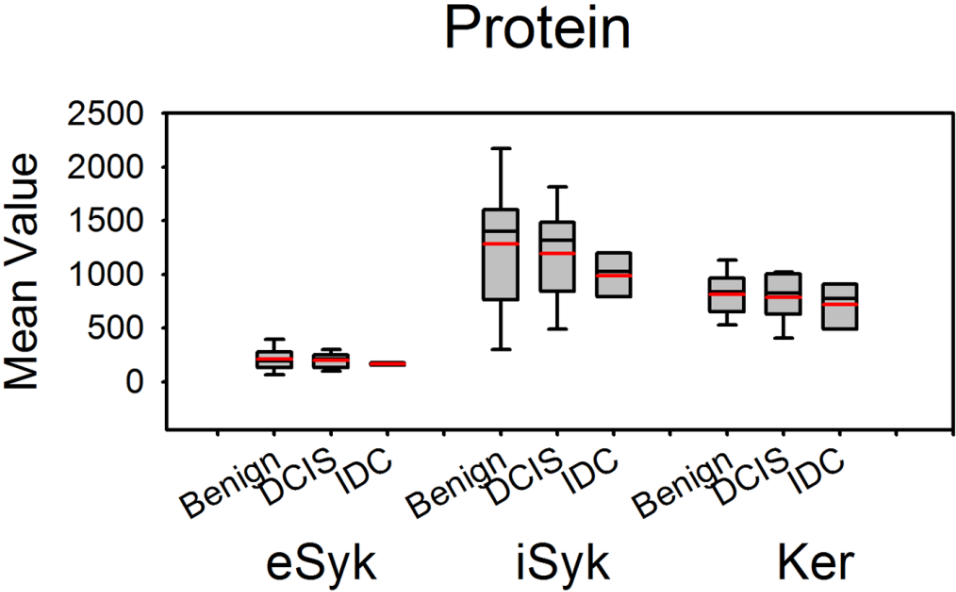

B

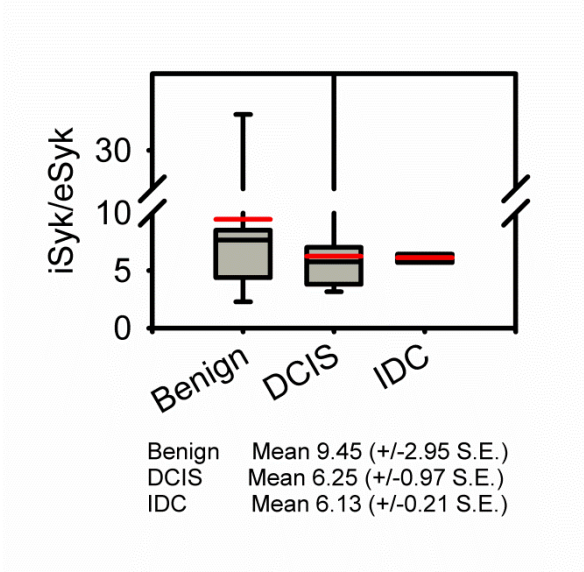

Supplement: Figure S2 — Quantitative immunofluorescence determination of protein. A. Mean intensity values were obtained as described in the Methods for each case by tissue type: Benign, DCIS, and IDC. Those means were averaged; the results for Syk in epithelial cells (eSyk), immune cells (eSyk) and keratin (Ker) are shown in the same graph to illustrate the relative staining for Syk in epithelial versus immune cells and as compared with keratin. Box plots were generated using Sigma Plot: red lines indicate the mean, black lines the median, and a 95%/5% range is indicated by whiskers. Boxes represent the 75%/25% range. B. The iSyk/eSyk protein ratios for benign, DCIS and IDC cases were graphed as a box plot. The mean and standard error (S.E.) are shown in the table below. (PDF) [file pone.0087610.s002.pdf]

Figure S 5

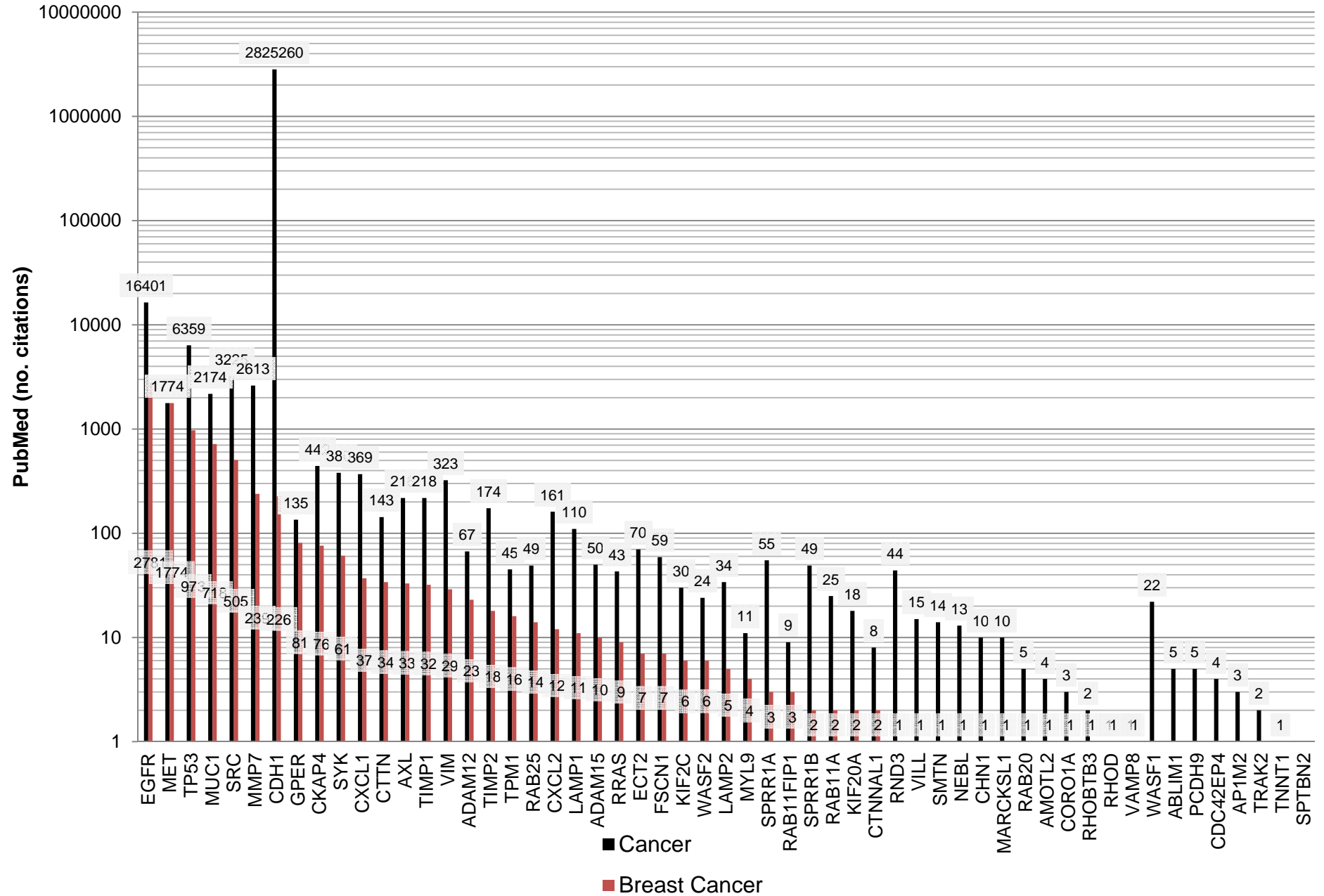

Supplement: Figure S5 — PubMed citations for members of the 55 Gene Set AND “Cancer” or members AND “Breast Cancer”. The number of citations for each gene was determined using PubMed for citations including both the gene symbol and the term “Cancer” (black bars) or “Breast Cancer” (red bars) and the results graphed. The results were sorted by Breast Cancer results with the most frequently cited breast cancer gene being EGFR. About half of the genes had been cited for breast cancer at least 5 times. 27 of the Genes were cited less than 5 times. One gene, SPTBN2 was cited 0 times for cancer and breast cancer. (PDF) [file pone.0087610.s005.pdf]
