# Supplementary material for: SYK Allelic Loss and the Role of Syk-Regulated Genes in Breast Cancer Survival
Source: PLoS One. 2014 Feb 11;9(2):e87610. doi: 10.1371/journal.pone.0087610 (PMC3921124; doi:10.1371/journal.pone.0087610)
Supplement: Figure S4 — Average copy number of members of the 55 Gene Set in IDC cases stratified by SYK copy number. A. Plot of average copy number (y-axis). B. Two gene plot of copy number for SYK and CTNNAL1 both of which are located on chromosome 9. (PDF) [file pone.0087610.s004.pdf]

Figure S 4

A

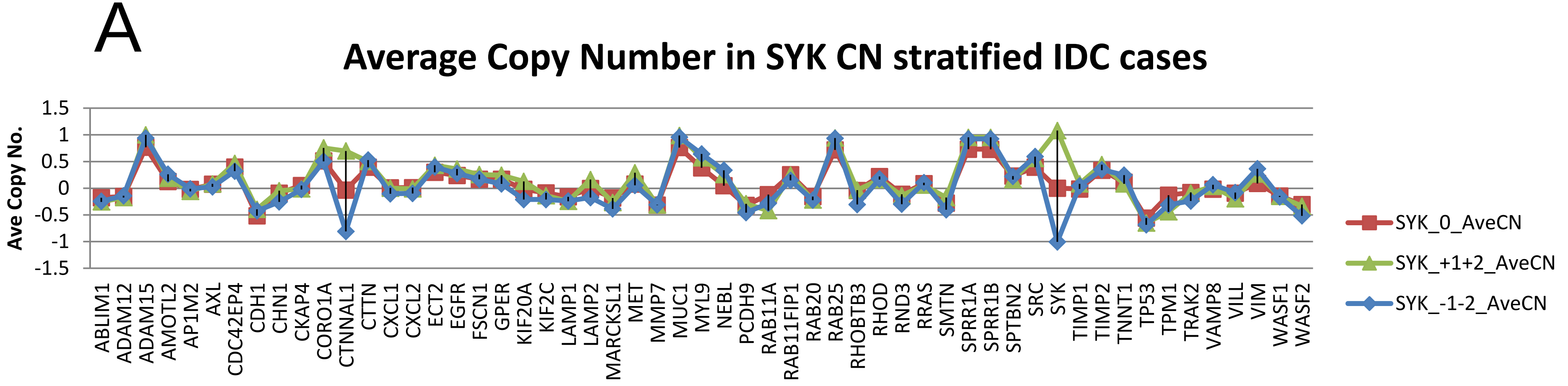

B

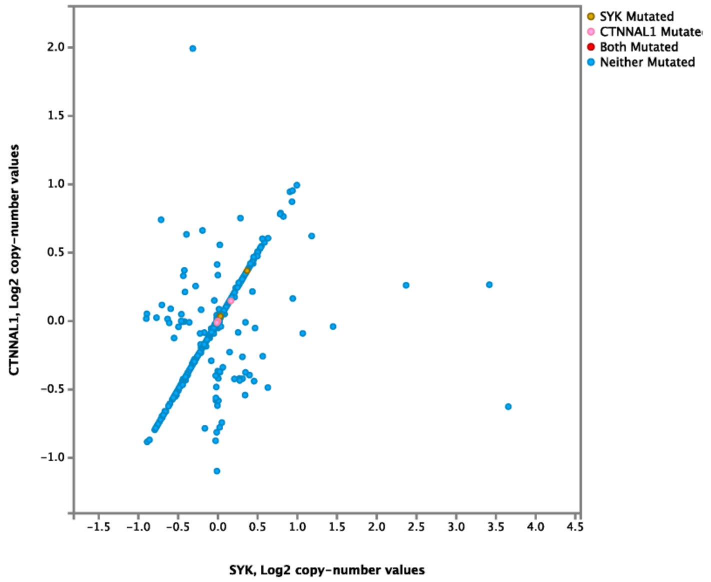

SYK: Location: 9q22 Annotation: Chromosome 9, NC_000009.11 (93564012..93660842)
CTNNAL1 (catenin (cadherin-associated protein), alpha-like 1, alpha-catulin: Location: 9q31.2 Annotation: Chromosome 9, NC_000009.11 (111704851..111775764, complement)
