# Supplementary material for: SYK Allelic Loss and the Role of Syk-Regulated Genes in Breast Cancer Survival
Source: PLoS One. 2014 Feb 11;9(2):e87610. doi: 10.1371/journal.pone.0087610 (PMC3921124; doi:10.1371/journal.pone.0087610)
Supplement: Table S1 — Summary of motility/invasion gene and mRNA data. Microarray data on significantly regulated mRNAs following SYK knockdown in MCF10A cells were obtained in a previous study [3]. Data on number of cases altered, % of cases altered and the average copy number gain or loss were determined from TCGA data as described in the Methods and Results . For number and % cases altered, copy number and mutational changes were evaluated using the cBioPortal software to determine and export this information for the 55 Gene Set. The table is organized by greatest mRNA fold changes to least with losses as numbers >1.5 fold change and gains as <0.67 fold change (VIM the greatest fold increase and SPRR1A the greatest fold decrease in mRNA expression after SYK knockdown). (PDF) [file pone.0087610.s006.pdf]

**Microarray study following *SYK* knockdown in MCF10A**							**TCGA study of CN/mut altered patient cases**				
**FINAL 55gene ID**	**FINAL 55gene GENE SYMBOL**	**Probe set**	**Description**	**mean of log intensities for collagen control**	**mean of log intensities for collagen SYK KD**	**FoldDiff (mRNA) [1]**	**Num Cases Altered**	**% Cases Altered**	**GENE_ID**	**COMMON**	**Ave CN gain/loss**
40	SPRR1A	213796_at	small proline-rich protein 1A	5.3513	1.23999	**17.28**	89	**12.80%**	6698	SPRR1A	**0.82**
40	SPRR1A	214549_x_at	small proline-rich protein 1A	6.63511	3.57669	**8.33**	89	**12.80%**	6698	SPRR1A	**0.82**
4	AMOTL2	203002_at	angiomotin like 2	4.05109	2.55416	**2.82**	10	**1.40%**	51421	AMOTL2	**0.17**
29	NEBL	203961_at	nebulette	4.04029	2.61005	**2.69**	23	**3.30%**	10529	NEBL	**0.18**
29	NEBL	203962_s_at	nebulette	4.44726	3.03057	**2.67**	23	**3.30%**	10529	NEBL	**0.18**
35	RHOBTB3	202975_s_at	Rho-related BTB domain containing 3	3.29064	2.05175	**2.36**	8	**1.10%**	22836	RHOBTB3	**-0.14**
32	RAB11FIP 1	219681_s_at	RAB11 family interacting protein 1 (class I)	3.33121	2.11184	**2.33**	104	**14.90%**	80223	RAB11FIP1	**0.20**
49	TPM1	206116_s_at	tropomyosin 1 (alpha)	4.9529	3.80533	**2.22**	7	**1.00%**	7168	TPM1	**-0.22**
35	RHOBTB3	202976_s_at	Rho-related BTB domain containing 3	2.96163	1.85266	**2.16**	8	**1.10%**	22836	RHOBTB3	**-0.14**
28	MYL9	201058_s_at	myosin, light chain 9, regulatory	2.44787	1.35916	**2.13**	19	**2.70%**	10398	MYL9	**0.47**
49	TPM1	210987_x_at	tropomyosin 1 (alpha)	5.3621	4.30889	**2.08**	7	**1.00%**	7168	TPM1	**-0.22**
49	TPM1	206117_at	tropomyosin 1 (alpha)	2.714	1.67104	**2.06**	7	**1.00%**	7168	TPM1	**-0.22**
34	RAB25	218186_at	RAB25, member RAS oncogene family	6.18144	5.18108	**2.00**	85	**12.20%**	57111	RAB25	**0.81**
49	TPM1	210986_s_at	tropomyosin 1 (alpha)	6.04476	5.07147	**1.96**	7	**1.00%**	7168	TPM1	**-0.22**

37	RND3	212724_at	Rho family GTPase 3	3.9234	2.97365	**1.93**	3	**0.40%**	390	RND3	**-0.17**
26	MMP7	204259_at	matrix metallopeptidase 7 (matrilysin, uterine)	1.85931	0.92773	**1.91**	14	**2.00%**	4316	MMP7	**-0.31**
27	MUC1	207847_s_at	mucin 1, cell surface associated	4.6449	3.73202	**1.88**	94	**13.50%**	4582	MUC1	**0.84**
11	CORO1A	209083_at	coronin, actin binding protein, 1A	2.77829	1.86761	**1.88**	37	**5.30%**	11151	CORO1A	**0.52**
46	TIMP2	203167_at	TIMP metallopeptidase inhibitor 2	3.60008	2.69077	**1.88**	47	**6.80%**	7077	TIMP2	**0.34**
27	MUC1	213693_s_at	mucin 1, cell surface associated	5.90508	5.00689	**1.86**	94	**13.50%**	4582	MUC1	**0.84**
36	RHOD	209885_at	ras homolog gene family, member D	5.77086	4.89576	**1.83**	33	**4.70%**	29984	RHOD	**0.18**
52	VILL	209950_s_at	villin-like	1.85609	1.00962	**1.80**	9	**1.30%**	50853	VILL	**-0.12**
5	AP1M2	65517_at	adaptor-related protein complex 1, mu 2 subunit	3.91307	3.08498	**1.78**	17	**2.40%**	10053	AP1M2	**-0.03**
5	AP1M2	218261_at	adaptor-related protein complex 1, mu 2 subunit	4.74543	3.91829	**1.77**	17	**2.40%**	10053	AP1M2	**-0.03**
29	NEBL	207279_s_at	nebulette	2.16513	1.35608	**1.75**	23	**3.30%**	10529	NEBL	**0.18**
9	CHN1	212624_s_at	chimerin (chimaerin) 1	4.32125	3.58034	**1.67**	6	**0.90%**	1123	CHN1	**-0.13**
44	SYK	207540_s_at	spleen tyrosine kinase	2.09222	1.35864	**1.66**	11	**1.60%**	6850	SYK	**-0.14**
36	RHOD	31846_at	ras homolog gene family, member D	6.65006	5.92047	**1.66**	33	**4.70%**	29984	RHOD	**0.18**
50	TRAK2	202124_s_at	trafficking protein, kinesin binding 2	2.90888	2.20315	**1.63**	12	**1.70%**	66008	TRAK2	**-0.12**
42	SPTBN2	205155_s_at	spectrin, beta, non-erythrocytic 2	2.7319	2.06609	**1.59**	42	**6.00%**	6712	SPTBN2	**0.20**

35	RHOBTB3	216048_s_at	Rho-related BTB domain containing 3	1.73529	1.08295	**1.57**	8	**1.10%**	22836	RHOBTB3	**-0.14**
30	PCDH9	219737_s_at	protocadherin 9	2.30688	1.67923	**1.55**	18	**2.60%**	5101	PCDH9	**-0.36**
31	RAB11A	200864_s_at	RAB11A, member RAS oncogene family	3.92712	3.3079	**1.54**	8	**1.10%**	8766	RAB11A	**-0.21**
1	ABLIM1	200965_s_at	actin binding LIM protein 1	4.80373	4.19058	**1.53**	8	**1.10%**	3983	ABLIM1	**-0.20**
7	CDC42EP4	214721_x_at	CDC42 effector protein (Rho GTPase binding) 4	2.86751	2.26813	**1.52**	56	**8.00%**	23580	CDC42EP4	**0.37**
50	TRAK2	202125_s_at	trafficking protein, kinesin binding 2	2.74738	2.15236	**1.51**	12	**1.70%**	66008	TRAK2	**-0.12**
51	VAMP8	202546_at	vesicle-associated membrane protein 8 (endobrevin)	6.43236	5.84249	**1.51**	7	**1.00%**	8673	VAMP8	**0.03**
19	GPER	210640_s_at	G protein-coupled estrogen receptor 1	1.66389	2.24987	**0.67**	11	**1.60%**	2852	GPER	**0.15**
2	ADAM12	213790_at	ADAM metallopeptidase domain 12 (meltrin alpha)	1.01558	1.61077	**0.66**	17	**2.40%**	8038	ADAM12	**-0.16**
20	KIF20A	218755_at	kinesin family member 20A	3.93348	4.52913	**0.66**	4	**0.60%**	10112	KIF20A	**-0.07**
24	MARCKSL 1	200644_at	MARCKS-like 1	2.2633	2.87432	**0.65**	7	**1.00%**	65108	MARCKSL1	**-0.24**
54	WASF1	204165_at	WAS protein family, member 1	2.45494	3.06884	**0.65**	20	**2.90%**	8936	WASF1	**-0.14**
17	EGFR	211607_x_at	epidermal growth factor receptor (erythroblastic leukemia viral (v-erb-b) oncogene homolog, avian)	2.16901	2.80621	**0.64**	21	**3.00%**	1956	EGFR	**0.27**

16	ECT2	219787_s_at	epithelial cell transforming sequence 2 oncogene	3.15335	3.79151	**0.64**	37	**5.30%**	1894	ECT2	**0.35**
21	KIF2C	211519_s_at	kinesin family member 2C	3.04305	3.68307	**0.64**	10	**1.40%**	11004	KIF2C	**-0.11**
17	EGFR	210984_x_at	epidermal growth factor receptor (erythroblastic leukemia viral (v-erb-b) oncogene homolog, avian)	2.33473	2.9983	**0.63**	21	**3.00%**	1956	EGFR	**0.27**
17	EGFR	201984_s_at	epidermal growth factor receptor (erythroblastic leukemia viral (v-erb-b) oncogene homolog, avian)	2.98141	3.65271	**0.63**	21	**3.00%**	1956	EGFR	**0.27**
21	KIF2C	209408_at	kinesin family member 2C	3.98417	4.68218	**0.62**	10	**1.40%**	11004	KIF2C	**-0.11**
10	CKAP4	200999_s_at	cytoskeleton-associated protein 4	3.94643	4.67124	**0.61**	4	**0.60%**	10970	CKAP4	**0.01**
25	MET	203510_at	met proto-oncogene (hepatocyte growth factor receptor)	4.11472	4.84654	**0.60**	9	**1.30%**	4233	MET	**0.10**
12	CTNNAL1	202468_s_at	catenin (cadherin-associated protein), alpha-like 1	4.07129	4.81079	**0.60**	8	**1.10%**	8727	CTNNAL1	**-0.16**
22	LAMP1	201551_s_at	lysosomal-associated membrane protein 1	3.33078	4.0833	**0.59**	29	**4.20%**	3916	LAMP1	**-0.19**
39	SMTN	209427_at	smoothelin	3.55693	4.31091	**0.59**	9	**1.30%**	6525	SMTN	**-0.31**

33	RAB20	219622_at	RAB20, member RAS oncogene family	2.85501	3.63536	**0.58**	28	**4.00%**	55647	RAB20	**-0.19**
38	RRAS	212647_at	related RAS viral (r-ras) oncogene homolog	3.32575	4.13953	**0.57**	18	**2.60%**	6237	RRAS	**0.09**
15	CXCL2	209774_x_at	chemokine (C-X-C motif) ligand 2	1.38434	2.20515	**0.57**	24	**3.40%**	2920	CXCL2	**-0.02**
3	ADAM15	217007_s_at	ADAM metallopeptidase domain 15	3.50458	4.32974	**0.56**	94	**13.50%**	8751	ADAM15	**0.84**
39	SMTN	207390_s_at	smoothelin	3.02705	3.86486	**0.56**	9	**1.30%**	6525	SMTN	**-0.31**
23	LAMP2	200821_at	lysosomal-associated membrane protein 2	2.99777	3.83674	**0.56**	9	**1.30%**	3920	LAMP2	**-0.03**
2	ADAM12	202952_s_at	ADAM metallopeptidase domain 12 (meltrin alpha)	1.31031	2.18956	**0.54**	17	**2.40%**	8038	ADAM12	**-0.16**
55	WASF2	221725_at	WAS protein family, member 2	3.80122	4.70557	**0.53**	5	**0.70%**	10163	WASF2	**-0.36**
17	EGFR	201983_s_at	epidermal growth factor receptor (erythroblastic leukemia viral (v-erb-b) oncogene homolog, avian)	4.65262	5.56341	**0.53**	21	**3.00%**	1956	EGFR	**0.27**
45	TIMP1	201666_at	TIMP metallopeptidase inhibitor 1	6.4029	7.3157	**0.53**	12	**1.70%**	7076	TIMP1	**0.02**
14	CXCL1	204470_at	chemokine (C-X-C motif) ligand 1 (melanoma growth stimulating activity, alpha)	1.24742	2.16559	**0.53**	23	**3.30%**	2919	CXCL1	**-0.02**

6	AXL	202686_s_at	AXL receptor tyrosine kinase	3.52507	4.47528	**0.52**	19	**2.70%**	558	AXL	**0.04**
18	FSCN1	201564_s_at	fascin homolog 1, actin-bundling protein (Strongylocentrotus purpuratus)	4.15038	5.26761	**0.46**	9	**1.30%**	6624	FSCN1	**0.18**
47	TNNT1	213201_s_at	troponin T type 1 (skeletal, slow)	2.15726	3.39845	**0.42**	23	**3.30%**	7138	TNNT1	**0.14**
18	FSCN1	210933_s_at	fascin homolog 1, actin-bundling protein (Strongylocentrotus purpuratus)	3.91259	5.16952	**0.42**	9	**1.30%**	6624	FSCN1	**0.18**
53	VIM	201426_s_at	vimentin	3.68115	5.4293	**0.30**	22	**3.20%**	7431	VIM	**0.21**

[1] **>=1.5 down-regulated genes; <= 0.67 up-regulated genes**
