# Supplementary material for: SYK Allelic Loss and the Role of Syk-Regulated Genes in Breast Cancer Survival
Source: PLoS One. 2014 Feb 11;9(2):e87610. doi: 10.1371/journal.pone.0087610 (PMC3921124; doi:10.1371/journal.pone.0087610)
Supplement: Table S2 — Summary of FISH, in situ , and methylation data for SYK in tumor epithelium. Cases were separated by whether they contained DCIS only, DCIS with IDC, or benign only tissues. SYK allelic loss, mRNA in situ, methylation, and Syk protein values where available are shown. Scores reflect the tissue types present in each case (slide) and available for analyses. For FISH results, the % DCIS was obtained from available pathology reports from the Histopathology and Tissue Shared Resource (second column). The number of FISH signals from the Syk and chromosome 9 centromere probes was determined microscopically by a licensed cytopathologist and their average ratio calculated which is shown in column 3 (J.B.). A cutoff point for “normal” versus “allelic loss” in DCIS tissues from each case was determined from Figure 1, as indicated. In situ hybridization results for SYK mRNA were obtained previously (Normal, Hyperplasia, DCIS, and Invasive in situ, columns 4–6 [4]. In the next to last set of data (columns 7–9), methylation results are shown. Raw Syk protein values were obtained from image analysis and are shown in the last columns (columns 10–12). (PDF) [file pone.0087610.s007.pdf]

Table S 2.

Summary of FISH, *in situ*, and methylation data for SYK in tumor epithelium.

Slide name	% DCIS	DCIS Allelic Loss [1]	Benign	DCIS	IDC	Benign	DCIS	IDC	Benign	DCIS	IDC
			SYK In situ [2]			**SYK Methylation** [3]			**Syk Protein** [4]		
**DCIS ONLY**											
38 **8081**	100	1.1		2.0			4.0			137.2	
38 **07405**	100	1.1	2.0	2.0			7.3		194.0	**273.1** [5]	
38 **2194**	100	0.9	3.0	2.0			N.D.		195.4	196.9	
38 **06830**	100	0.8	3.0	3.0			N.D.		93.4	**123.7**	
38 06768	100	0.7	3.0			5.8	**9.3** [6]		206.9		
38 **10095**	100	1.0	3.0	2.0			**10.3**		173.3	208.8	
38 1855	100	0.9	3.0	2.0			**9.8**		145.1	**80.3**	
38 **4205**	100	0.9		0.0			**9.1**		202.5	192.3	
**DCIS with IDC**											
39 7038	60	0.9	3.0		0.0		N.D.		N.D.		
36 2059		0.9	3.0	2.0	2.5		N.D.	3.9	N.D.		
38 **02422**	70	0.9		0.0	0.0		**9.7**	3.4	281.0	**224.1**	**174.7**
36 10462		0.9	1.0	3.0	2.0		**10.7**	5.0	N.D.		
39 **04168**	70	0.8		2.0			4.3		N.D.	246.0	
38 10443	50	0.8	3.0	0.0			**11.2**		N.D.		
39 **06013**	98	**0.5** [7]|	3.0	3.0			7.0		421.5	**214.5**	
38 **11203**	70	**0.6**	3.0	2.0	0.0		2.2			308.3	350.0
39 **08204**	70	**0.3**	3.0	2.0			5.3	**13.5**	279.6	253.8	**174.8**
39 **07489**	50	**0.6**		2.0	0.0		4.5	**11.1**		121.3	**150.6**
36 **6362**		**0.5**	3.0	2.5	2.5			7.7		216.5	**168.8**

**Benign ONLY**					
35 870	benign	0.8	3.5	6.0	47.6
35 4850	benign	0.8	3.5	1.7	118.8
36 11964	benign	0.7	3.0	7.3	
36 10605	benign	0.8	3.0		
36 3757	benign	0.7			

[1] Ratio of Syk/ Chromosome 9 Centromere marker, Average of 30 cells, cut off of 0.6

[2] from Moroni et al., (2004) Progressive loss of Syk and abnormal proliferation in breast cancer cells. Cancer Res 64: 7346-7354.

[3] Pyrosequencing, result is average of 4 CpG's, cut off above normal breast control + 1 S.D.

[4] Immunofluorescence staining for epithelial Syk, average intensity (bold is P < 0.05 for adjacent value).

[5] Bold indicates protein staining intensity significantly different from tissue to left, P < 0.05.

[6] Bold indicates methylation value in the positive range (see *Methods*).

[7] Bold indicates allelic loss (see *Methods*).
