# Supplementary material for: SYK Allelic Loss and the Role of Syk-Regulated Genes in Breast Cancer Survival
Source: PLoS One. 2014 Feb 11;9(2):e87610. doi: 10.1371/journal.pone.0087610 (PMC3921124; doi:10.1371/journal.pone.0087610)
Supplement: Table S3 — Functional Annotation Table for the 55 Gene Set Members. A functional gene annotation table was generated using DAVID (http://david.abcc.ncifcrf.gov/) to illustrate the groups of genes involved in motility- and invasion-related activities. Some of the important activities are highlighted. (PDF) [file pone.0087610.s008.pdf]

Table S 3

Category	Term	Count	%	PValue	Genes	List Total	Pop Hits	Pop Total	Fold Enrichment	Bonferroni	Benjamini	FDR
GOTERM_BP_FAT	prenylation	6	1.010101	7.55E-05	RND3, RRAS, RAB11A, RAB25, RHOD, RAB20	55	156	19235	13.45104895	0.010593133	0.001773366	0.089189
GOTERM_BP_FAT	short sequence motif:Effector region	6	1.010101	7.12E-06	RND3, RRAS, RAB11A, RAB25, RHOD, RAB20	55	95	19113	21.94794258	0.002338387	0.002338387	0.009642
GOTERM_BP_FAT	IPR001806:Ras GTPase	6	1.010101	6.81E-05	RND3, RRAS, RAB11A, RAB25, RHOD, RAB20	54	135	16659	13.71111111	0.00956178	0.004792373	0.080467
GOTERM_BP_FAT	GO:0030674~protein binding, bridging	5	0.841751	3.06E-04	SPRR1A, SPRR1B, FSCN1, CHN1, SRC	46	94	12983	15.0127197	0.034573947	0.008757867	0.34791
GOTERM_BP_FAT	nucleotide-binding	14	2.356902	6.26E-04	EGFR, MET, AXL, SRC, RND3, KIF2C, RAB11A, RRAS, RAB25, RHOD, RAB20, SYK, KIF20A, RHOBTB3	55	1686	19235	2.904022431	0.084471623	0.009758069	0.736711
GOTERM_BP_FAT	IPR001245:Tyrosine protein kinase	5	0.841751	5.36E-04	EGFR, MET, AXL, SRC, SYK	54	118	16659	13.0720339	0.072793149	0.015002051	0.631237
GOTERM_BP_FAT	transmembrane protein	8	1.346801	0.002025	EGFR, MUC1, LAMP1, LAMP2, GPER, MET, AXL, CDH1	55	642	19235	4.357972246	0.248649508	0.028183524	2.366822
GOTERM_BP_FAT	lipid moiety-binding region:S-geranylgeranyl cysteine	5	0.841751	1.97E-04	RRAS, RAB11A, RAB25, RHOD, RAB20	55	102	19113	17.03475936	0.06275721	0.031886995	0.266597
GOTERM_BP_FAT	GO:0005200~structural constituent of cytoskeleton	4	0.673401	0.002124	VIM, SPTBN2, TPM1, VILL	46	74	12983	15.25616921	0.216937466	0.034331503	2.393109
GOTERM_BP_FAT	GO:0032555~purine ribonucleotide binding	15	2.525253	0.002759	EGFR, MET, TP53, AXL, SRC, RND3, KIF2C, RAB11A, RRAS, RAB25, RHOD, RAB20, SYK, KIF20A, RHOBTB3	46	1836	12983	2.305875249	0.272176835	0.034683918	3.097811
GOTERM_BP_FAT	GO:0032553~ribonucleotide binding	15	2.525253	0.002759	EGFR, MET, TP53, AXL, SRC, RND3, KIF2C, RAB11A, RRAS, RAB25, RHOD, RAB20, SYK, KIF20A, RHOBTB3	46	1836	12983	2.305875249	0.272176835	0.034683918	3.097811
GOTERM_BP_FAT	GO:0004713~protein tyrosine kinase activity	5	0.841751	0.002553	EGFR, MET, AXL, SRC, SYK	46	166	12983	8.501178628	0.254741681	0.036085854	2.870328
GOTERM_BP_FAT	transforming protein	3	0.505051	0.004097	AXL, RRAS, SRC	55	34	19235	30.85828877	0.439469288	0.037856255	4.734355
GOTERM_BP_FAT	GO:0030029~actin filament-based process	7	1.178451	2.48E-04	CXCL1, RND3, CORO1A, WASF1, WASF2, FSCN1, TPM1	51	241	13528	7.704499227	0.178971791	0.038671947	0.379304
GOTERM_BP_FAT	GO:0032970~regulation of actin filament-based process	5	0.841751	3.63E-04	CORO1A, SPTBN2, TPM1, VILL, NEBL	51	92	13528	14.41602728	0.251097881	0.040465216	0.555673
GOTERM_BP_FAT	GO:0032956~regulation of actin cytoskeleton organization	5	0.841751	3.20E-04	CORO1A, SPTBN2, TPM1, VILL, NEBL	51	89	13528	14.90196078	0.224870036	0.041565538	0.489684
GOTERM_BP_FAT	hsa05219:Bladder cancer	3	0.505051	0.015099	EGFR, TP53, CDH1	24	42	5085	15.13392857	0.539709022	0.10492015	13.68583
GOTERM_BP_FAT	hsa05213:Endometrial cancer	3	0.505051	0.022626	EGFR, TP53, CDH1	24	52	5085	12.22355769	0.688755723	0.121629118	19.86013
GOTERM_BP_FAT	GO:0004714~transmembrane receptor protein tyrosine kinase activity	3	0.505051	0.02252	EGFR, MET, AXL	46	67	12983	12.637573	0.927156449	0.160231834	22.85297
GOTERM_BP_FAT	GO:0003924~GTPase activity	4	0.673401	0.036443	RND3, RRAS, RAB11A, RHOD	46	211	12983	5.350504842	0.98600706	0.222078417	34.48341
GOTERM_BP_FAT	GO:0031988~membrane-bounded vesicle	7	1.178451	0.026997	EGFR, LAMP1, CORO1A, LAMP2, RAB11A, RAB11FIP1, TIMP1	53	568	12782	2.972163168	0.994330407	0.227892812	28.8127
GOTERM_BP_FAT	GO:0044459~plasma membrane part	16	2.693603	0.026041	EGFR, MUC1, AP1M2, GPER, MET, AXL, CDH1, TPM1, AMOTL2, SRC, LAMP1, CORO1A, RRAS, RAB25, RHOD, SYK	53	2203	12782	1.751573754	0.993173047	0.230848408	27.93854
GOTERM_BP_FAT	GO:0001726~ruffle	3	0.505051	0.030333	CTTN, WASF2, TPM1	53	67	12782	10.79864827	0.99703759	0.242117022	31.7849
GOTERM_BP_FAT	IPR002000:Lysosome-associated membrane glycoprotein (Lamp)/CD68	2	0.3367	0.015808	LAMP1, LAMP2	54	5	16659	123.4	0.89426057	0.24485627	17.159
GOTERM_BP_FAT	GO:0005794~Golgi apparatus	9	1.515152	0.023583	RND3, AP1M2, VAMP8, GPER, RAB11A, CDH1, RAB20, KIF20A, RHOBTB3	53	872	12782	2.489137961	0.989007721	0.245658531	25.64771
GOTERM_BP_FAT	GO:0031267~small GTPase binding	3	0.505051	0.047779	CDC42EP4, ECT2, RHOBTB3	46	101	12983	8.383340508	0.996412033	0.256454047	42.74593
GOTERM_BP_FAT	short sequence motif:Cysteine switch	3	0.505051	0.006569	MMP7, ADAM12, ADAM15	55	43	19113	24.2448203	0.885641292	0.303301503	8.544089
GOTERM_BP_FAT	region of interest:2 X 12 AA approximate repeats	2	0.3367	0.005643	SPRR1A, SPRR1B	55	2	19113	347.5090909	0.844596254	0.310884058	7.381503
GOTERM_BP_FAT	region of interest:6 X 8 AA approximate tandem repeats	2	0.3367	0.005643	SPRR1A, SPRR1B	55	2	19113	347.5090909	0.844596254	0.310884058	7.381503
GOTERM_BP_FAT	glycosylation site:N-linked (GlcNAc...) (polylactosaminoglycan)	2	0.3367	0.005643	LAMP1, LAMP2	55	2	19113	347.5090909	0.844596254	0.310884058	7.381503
GOTERM_BP_FAT	SM00153:VHP	2	0.3367	0.031297	ABLIM1, VILL	37	8	9079	61.34459459	0.701290007	0.331527954	24.97581
GOTERM_BP_FAT	PIRSF002462:lysosome-associated membrane protein	2	0.3367	0.012121	LAMP1, LAMP2	31	3	7396	159.0537634	0.370868686	0.370868686	10.43555
GOTERM_BP_FAT	IPR002473:Small chemokine, C-X-C/Interleukin 8	2	0.3367	0.043648	CXCL1, CXCL2	54	14	16659	44.07142857	0.998150204	0.408083668	40.97649

Table S 3

GOTERM_BP_FAT	IPR001089:Small chemokine, C-X-C	2	0.3367	0.040593	CXCL1, CXCL2	54	13	16659	47.46153846	0.997099949	0.412091683	38.7104
GOTERM_BP_FAT	metal ion-binding site:Zinc; via amide nitrogen and carbonyl oxygen; shared with metalloproteinase partner	2	0.3367	0.011254	TIMP2, TIMP1	55	4	19113	173.7545455	0.975854387	0.412541908	14.21883
GOTERM_BP_FAT	GO:0032535~regulation of cellular component size	5	0.841751	0.017635	CORO1A, TP53, SPTBN2, VILL, NEBL	51	271	13528	4.894001881	0.999999293	0.44573532	23.88565
GOTERM_BP_FAT	GO:0040012~regulation of locomotion	4	0.673401	0.033887	EGFR, CORO1A, RRAS, TPM1	51	192	13528	5.526143791	1	0.54345107	41.0714
GOTERM_BP_FAT	GO:0007167~enzyme linked receptor protein signaling pathway	5	0.841751	0.03718	EGFR, MET, AXL, SRC, SYK	51	342	13528	3.877995643	1	0.547818369	44.07798
GOTERM_BP_FAT	GO:0046777~protein amino acid autophosphorylation	3	0.505051	0.039344	EGFR, MET, SYK	51	85	13528	9.361937716	1	0.550119758	45.97565
GOTERM_CC_FAT	GO:0015629~actin cytoskeleton	13	2.188552	5.77E-10	ABLIM1, CTNNAL1, WASF1, FSCN1, WASF2, CDH1, TPM1, VILL, MYL9, CORO1A, SMTN, TNNT1, SPTBN2	53	269	12782	11.65504664	1.09E-07	1.09E-07	7.16E-07
GOTERM_CC_FAT	GO:0005856~cytoskeleton	20	3.367003	8.86E-07	ABLIM1, CTNNAL1, WASF1, VIM, FSCN1, WASF2, CDH1, TPM1, VILL, MYL9, KIF2C, CORO1A, CTTN, TNNT1, SMTN, SPRR1A, SPRR1B, SPTBN2, CDC42EP4, KIF20A	53	1381	12782	3.492683727	1.67E-04	8.37E-05	0.0011
GOTERM_CC_FAT	Proto-oncogene	5	0.841751	0.003945	EGFR, MET, AXL, ECT2, SRC	55	230	19235	7.602766798	0.427279987	0.039029251	4.562487
GOTERM_CC_FAT	SM00219:TyrKc	5	0.841751	0.001158	EGFR, MET, AXL, SRC, SYK	37	118	9079	10.39738891	0.043091882	0.043091882	1.042101
GOTERM_CC_FAT	phosphoprotein	31	5.218855	0.005851	ABLIM1, CTNNAL1, AP1M2, MARCKSL1, VIM, WASF2, CDH1, AMOTL2, SRC, MYL9, KIF2C, CTTN, CDC42EP4, SYK, MUC1, EGFR, CKAP4, FSCN1, MET, AXL, TP53, ECT2, CORO1A, SMTN, TNNT1, VAMP8, SPTBN2, ADAM12, RAB11FIP1, ADAM15, KIF20A	55	7263	19235	1.492708998	0.562791134	0.044923244	6.697131
GOTERM_CC_FAT	hsa05218:Melanoma	4	0.673401	0.003781	EGFR, MET, TP53, CDH1	24	71	5085	11.93661972	0.175693951	0.062374315	3.598633
GOTERM_CC_FAT	muscle protein	3	0.505051	0.012339	TNNT1, TPM1, MYL9	55	60	19235	17.48636364	0.826336121	0.079983343	13.64275
GOTERM_CC_FAT	cytoplasm	17	2.861953	0.017909	ABLIM1, CTNNAL1, MUC1, WASF1, FSCN1, WASF2, TP53, TPM1, KIF2C, CORO1A, CTTN, SMTN, TRAK2, SPRR1A, SPRR1B, SPTBN2, CDC42EP4	55	3332	19235	1.78432282	0.921763999	0.104867665	19.22375
GOTERM_CC_FAT	hsa04810:Regulation of actin cytoskeleton	5	0.841751	0.014612	EGFR, WASF1, WASF2, RRAS, MYL9	24	215	5085	4.927325581	0.52798034	0.117611144	13.27288
GOTERM_CC_FAT	GO:0051015~actin filament binding	3	0.505051	0.014471	EGFR, CORO1A, FSCN1	46	53	12983	15.97579984	0.812942718	0.120981435	15.29897
GOTERM_CC_FAT	GO:0010576~metalloenzyme regulator activity	2	0.3367	0.034137	TIMP2, TIMP1	46	10	12983	56.44782609	0.981579527	0.220921358	32.67489
GOTERM_CC_FAT	GO:0031410~cytoplasmic vesicle	8	1.346801	0.014619	EGFR, LAMP1, CORO1A, LAMP2, AP1M2, RAB11A, RAB11FIP1, TIMP1	53	642	12782	3.005231294	0.93817305	0.242961821	16.713
GOTERM_CC_FAT	PIRSF001636:metalloproteinase inhibitor	2	0.3367	0.01613	TIMP2, TIMP1	31	4	7396	119.2903226	0.460940276	0.265793133	13.66705
GOTERM_CC_FAT	GO:0031982~vesicle	8	1.346801	0.018097	EGFR, LAMP1, CORO1A, LAMP2, AP1M2, RAB11A, RAB11FIP1, TIMP1	53	670	12782	2.879639538	0.968307069	0.269325661	20.29064
GOTERM_CC_FAT	GO:0012506~vesicle membrane	4	0.673401	0.023428	EGFR, CORO1A, LAMP2, RAB11FIP1	53	151	12782	6.388604273	0.98867225	0.273876888	25.5007
GOTERM_CC_FAT	GO:0042995~cell projection	8	1.346801	0.021974	CORO1A, CTTN, WASF1, MET, WASF2, VIM, FSCN1, TPM1	53	697	12782	2.768089656	0.984995464	0.276048574	24.112
GOTERM_CC_FAT	propeptide:Removed in mature form	5	0.841751	0.003973	RND3, RRAS, RAB11A, RAB25, RHOD	55	229	19113	7.587534736	0.730089761	0.279216494	5.251371
GOTERM_CC_FAT	region of interest:First lumenal domain	2	0.3367	0.005643	LAMP1, LAMP2	55	2	19113	347.5090909	0.844596254	0.310884058	7.381503
GOTERM_CC_FAT	IPR003128:Villin headpiece	2	0.3367	0.025175	ABLIM1, VILL	54	8	16659	77.125	0.972543935	0.329319984	26.00868
GOTERM_CC_FAT	PIRSF038481:Wiskott-Aldrich syndrome proteins	2	0.3367	0.012121	WASF1, WASF2	31	3	7396	159.0537634	0.370868686	0.370868686	10.43555
GOTERM_CC_FAT	IPR006025:Peptidase M, neutral zinc metallopeptidases, zinc-binding site	3	0.505051	0.037445	MMP7, ADAM12, ADAM15	54	96	16659	9.640625	0.99539746	0.41615206	36.29208
GOTERM_CC_FAT	GO:0044057~regulation of system process	5	0.841751	0.027003	EGFR, TNNT1, RAB11A, TPM1, MYL9	51	309	13528	4.292150517	1	0.483303843	34.29
GOTERM_CC_FAT	GO:0051674~localization of cell	5	0.841751	0.026448	CORO1A, MET, WASF2, SRC, SYK	51	307	13528	4.32011241	0.999999999	0.486626927	33.71279
GOTERM_MF_FAT	GO:0003779~actin binding	11	1.851852	1.27E-07	EGFR, ABLIM1, CORO1A, SMTN, WASF1, WASF2, FSCN1, SPTBN2, TPM1, VILL, NEBL	46	326	12983	9.523406242	1.46E-05	1.46E-05	1.45E-04
GOTERM_MF_FAT	actin-binding	9	1.515152	4.13E-07	ABLIM1, CORO1A, WASF1, WASF2, FSCN1, SPTBN2, TPM1, VILL, NEBL	55	247	19235	12.74309901	5.82E-05	5.82E-05	4.87E-04

Table S 3

GOTERM_MF_FAT	GO:0007010~cytoskeleton organization	10	1.683502	2.67E-05	ABLIM1, CXCL1, KIF2C, RND3, CORO1A, WASF1, WASF2, FSCN1, TPM1, VILL	51	436	13528	6.083828027	0.021066885	0.010589511	0.041024
GOTERM_MF_FAT	GO:0007264~small GTPase mediated signal transduction	9	1.515152	1.44E-05	RND3, WASF2, RRAS, RAB11A, RAB25, RHOD, CDC42EP4, RAB20, SRC	51	305	13528	7.827193828	0.01135783	0.01135783	0.022011
GOTERM_MF_FAT	phosphotransferase	5	0.841751	0.002433	EGFR, MET, AXL, SRC, SYK	55	201	19235	8.699683401	0.290743791	0.0282223	2.837319
GOTERM_MF_FAT	erythropoiesis	2	0.3367	0.005607	TIMP2, TIMP1	55	2	19235	349.7272727	0.547431357	0.045565466	6.426817
GOTERM_MF_FAT	GO:0007265~Ras protein signal transduction	5	0.841751	6.00E-04	WASF2, RRAS, RHOD, CDC42EP4, SRC	51	105	13528	12.63118581	0.379731314	0.057953193	0.916178
GOTERM_MF_FAT	GO:0022610~biological adhesion	10	1.683502	9.47E-04	EGFR, CTNNAL1, RND3, CORO1A, PCDH9, CDH1, ADAM12, SRC, ADAM15, SYK	51	701	13528	3.783950099	0.529582578	0.072640042	1.442804
GOTERM_MF_FAT	GO:0016192~vesicle-mediated transport	9	1.515152	0.001137	CORO1A, AP1M2, VAMP8, MARCKSL1, WASF2, SPTBN2, SRC, KIF20A, RHOBTB3	51	576	13528	4.144607843	0.595548594	0.072660279	1.729353
GOTERM_MF_FAT	protein transport	6	1.010101	0.011449	AP1M2, RAB11A, RAB25, RAB11FIP1, RAB20, KIF20A	55	485	19235	4.326522962	0.802829231	0.077976211	12.71932
GOTERM_MF_FAT	GO:0030832~regulation of actin filament length	4	0.673401	0.001614	CORO1A, SPTBN2, VILL, NEBL	51	63	13528	16.84158108	0.723602239	0.087758955	2.447666
GOTERM_MF_FAT	golgi apparatus	6	1.010101	0.024256	RND3, AP1M2, GPER, RAB20, KIF20A, RHOBTB3	55	588	19235	3.56864564	0.968639977	0.129327045	25.17989
GOTERM_MF_FAT	nucleotide phosphate-binding region:GTP	6	1.010101	0.001443	RND3, RRAS, RAB11A, RAB25, RHOD, RAB20	55	295	19113	7.06798151	0.378178368	0.146463806	1.937839
GOTERM_MF_FAT	GO:0005886~plasma membrane	25	4.208754	0.008199	CTNNAL1, AP1M2, MARCKSL1, VIM, CDH1, AMOTL2, TPM1, SRC, TRAK2, RRAS, RAB11A, RAB25, RHOD, SYK, EGFR, MUC1, GPER, FSCN1, MET, AXL, PCDH9, LAMP1, CORO1A, LAMP2, ADAM12	53	3777	12782	1.596305344	0.789033555	0.158774172	9.718438
GOTERM_MF_FAT	GO:0008191~metalloendopeptidase inhibitor activity	2	0.3367	0.034137	TIMP2, TIMP1	46	10	12983	56.44782609	0.981579527	0.220921358	32.67489
GOTERM_MF_FAT	GO:0030670~phagocytic vesicle membrane	2	0.3367	0.024167	CORO1A, RAB11FIP1	53	6	12782	80.38993711	0.99018338	0.226532033	26.19825
GOTERM_MF_FAT	GO:0030659~cytoplasmic vesicle membrane	4	0.673401	0.018871	EGFR, CORO1A, LAMP2, RAB11FIP1	53	139	12782	6.940138455	0.972695084	0.259224284	21.0673
GOTERM_MF_FAT	GO:0016044~membrane organization	6	1.010101	0.012943	EGFR, CORO1A, AP1M2, VAMP8, WASF2, TP53	51	381	13528	4.177242551	0.999968648	0.404594085	18.11445
GOTERM_MF_FAT	GO:0016337~cell-cell adhesion	5	0.841751	0.018728	EGFR, PCDH9, CDH1, SRC, SYK	51	276	13528	4.805342427	0.999999709	0.452264811	25.17476
GOTERM_MF_FAT	GO:0051494~negative regulation of cytoskeleton organization	3	0.505051	0.01755	CORO1A, SPTBN2, VILL	51	55	13528	14.4684492	0.999999243	0.458161039	23.78493
GOTERM_MF_FAT	GO:0015031~protein transport	8	1.346801	0.021139	AP1M2, TRAK2, TP53, RAB11A, RAB25, RAB11FIP1, RAB20, KIF20A	51	762	13528	2.784828367	0.999999959	0.480096902	27.94534
