# Supplementary material for: SYK Allelic Loss and the Role of Syk-Regulated Genes in Breast Cancer Survival
Source: PLoS One. 2014 Feb 11;9(2):e87610. doi: 10.1371/journal.pone.0087610 (PMC3921124; doi:10.1371/journal.pone.0087610)
Supplement: Table S4 — Protein/phosphoprotein results from immune depleted all types or IDC only cases when queried using cBioPortal for the 55 Gene Set. Immune depleted invasive breast cancer cases (800 cases) or immune depleted IDC only cases (565 cases) were queried using the 55 Gene Set for mutation and copy number changes. The significant protein and phosphoprotein changes comparing the altered versus nonaltered cases were extracted using cBioPortal. (PDF) [file pone.0087610.s009.pdf]

Table S 4

All Immune Depleted

Protein	Residue	Unaltered	Altered	p-value
AKT1/ AKT2/ AKT3	pT308	-0.27	0.07	0.003
AKT1S1	pT246	-0.29	0.04	0.007
ARAF	pS299	-0.28	0.1	3.07E-04
ASNS		-0.5	0.1	2.96E-08
BCL2		0.37	-0.08	4.49E-05
BCL2L11		0.22	-0.05	0.023
CASP7	cleavedD198	-0.36	-0.09	0.001
CAV1		0.21	-0.04	0.045
CCNB1		-0.42	0.12	2.63E-05
CCNE1		-0.43	0.04	1.56E-06
CDH3		-0.36	0.03	3.33E-04
CDK1		-0.36	0.08	2.50E-04
CDKN1B	pT198	-0.3	0.01	0.002
CHEK2	pT68	-0.29	0.07	0.004
CHEK2		-0.2	0.07	0.023
EEF2		-0.27	0.07	0.006
EEF2K		0.36	-0.01	0.004
EGFR		-0.3	0.01	7.23E-04
EIF4EBP1	pT70	-0.34	0.12	6.25E-05
EIF4EBP1	pS65	-0.29	0.06	0.004
EIF4EBP1	pT37	-0.25	0.08	0.013
EIF4EBP1		-0.19	0.09	0.016
ESR1	pS118	0.4	-0.01	0.002

IDC Immune Depleted

Protein	Residue	Unaltered	Altered	p-value
AKT1/AKT2/AKT3	pT308	-0.3	0.03	0.009
AKT1S1	pT246	-0.27	0.04	0.027
ARAF	pS299	-0.26	0.08	0.002
ASNS		-0.48	0.16	1.06E-07
BCL2		0.34	-0.07	0.001
BCL2L11		0.28	-0.03	0.02
CASP7	cleavedD198	-0.43	-0.03	9.28E-06
CAV1		0.27	-0.1	0.006
CCNB1		-0.45	0.16	1.35E-05
CCNE1		-0.39	0.08	3.05E-05
CDH1		0.36	0.16	0.043
CDH3		-0.38	0.06	5.74E-04
CDK1		-0.36	0.08	7.78E-04
CDKN1B	pT198	-0.3	0.04	7.30E-04
CDKN1B		0.2	-0.13	0.016
CHEK2	pT68	-0.31	0.11	0.003
CHEK2		-0.27	0.11	0.002
COL6A1		0.19	-0.12	0.032
DVL3		-0.09	0.16	0.043
EEF2		-0.32	0.12	0.002
EEF2K		0.32	-0.04	0.011
EGFR		-0.25	-0.01	0.023
EIF4EBP1	pT70	-0.39	0.13	6.68E-07
EIF4EBP1	pS65	-0.38	0.05	9.32E-05
EIF4EBP1	pT37	-0.32	0.07	0.004
EIF4EBP1		-0.22	0.07	0.015
ESR1	pS118	0.37	-0.03	0.003

Table S 4

ESR1		0.54	-0.02	3.13E-08	ESR1		0.54	-0.05	1.12E-07
GATA3		0.56	-0.02	6.98E-08	GATA3		0.54	-0.03	1.19E-06
INPP4B		0.34	0.03	0.008	INPP4B		0.39	0.02	0.003
IRS1		0.25	-0.06	0.026	IRS1		0.25	-0.07	0.032
MAP2K1	pS217	-0.21	0.05	0.033	MAP2K1	pS217	-0.29	0.06	0.008
MAPK14		0.16	-0.12	0.029					
MAPK9		0.32	-0.04	0.002	MAPK9		0.28	-0.09	0.005
NFKB1	pS536	-0.29	0.06	0.006	NFKB1	pS536	-0.27	0.11	0.007
PARK7		0.24	-0.08	0.003	PARK7		0.18	-0.16	0.006
PDK1	pS241	0.27	0.01	0.029					
PGR		0.43	-0.05	2.13E-04	PGR		0.41	-0.04	0.001
RAD50		0.32	-0.05	8.17E-04	RAD50		0.28	-0.03	0.012
					RPS6	pS235	-0.26	0.06	0.016
					RPS6	pS240	-0.22	0.09	0.02
					RPS6		-0.21	0.09	0.021
SCD1		-0.19	0.08	0.033	SCD1		-0.24	0.15	0.008
SMAD3		0.25	-0.02	0.037	SMAD3		0.29	-0.08	0.007
SRC	pY416	-0.31	0.04	0.004	SRC	pY416	-0.34	0.03	0.006
STAT5A		0.19	-0.1	0.013	TP53		-0.26	0.11	0.005
TP53		-0.31	0.08	0.001					
TP53BP1		0.28	0	0.024					
TSC2		0.35	0.01	0.004	TSC2		0.33	0.01	0.017
