# Supplementary material for: SYK Allelic Loss and the Role of Syk-Regulated Genes in Breast Cancer Survival
Source: PLoS One. 2014 Feb 11;9(2):e87610. doi: 10.1371/journal.pone.0087610 (PMC3921124; doi:10.1371/journal.pone.0087610)

Figure S 3

A

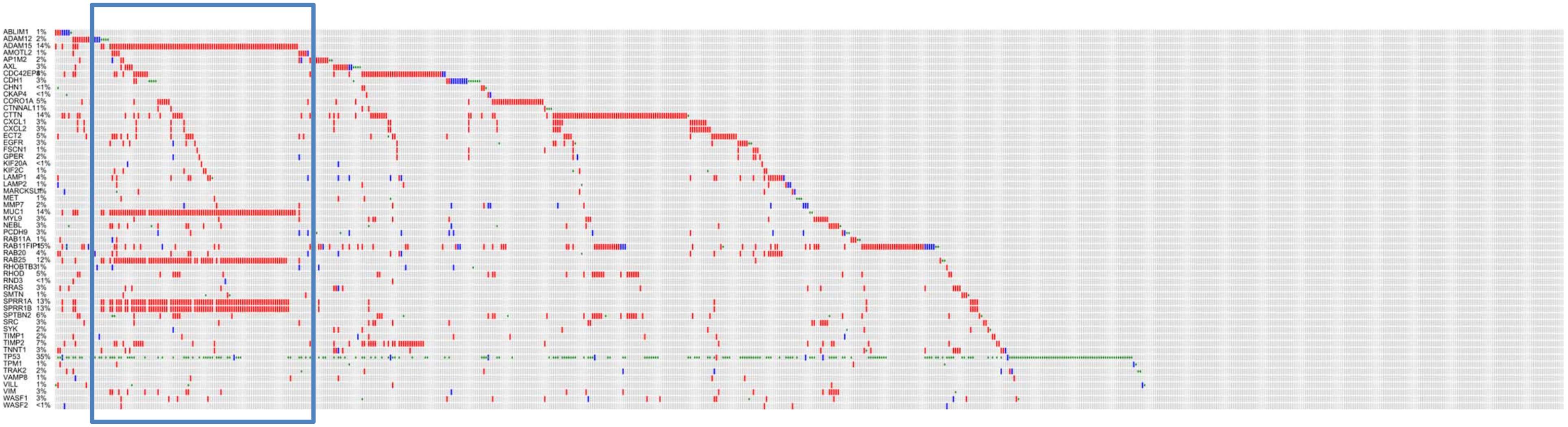

B

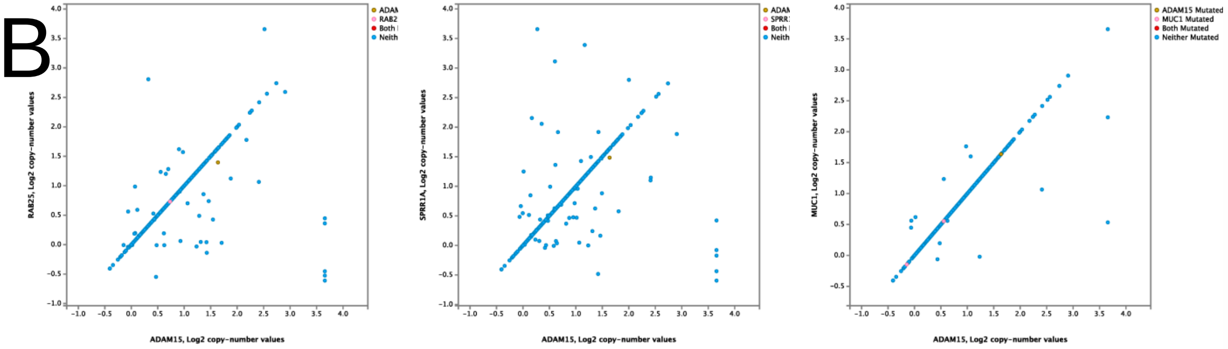

Gene Set / Pathway is altered in 15.7% of all cases.

Breast Invasive Carcinoma (TCGA, Provisional) / User-defined Case List: (696) / User-defined List / 5 genes

C

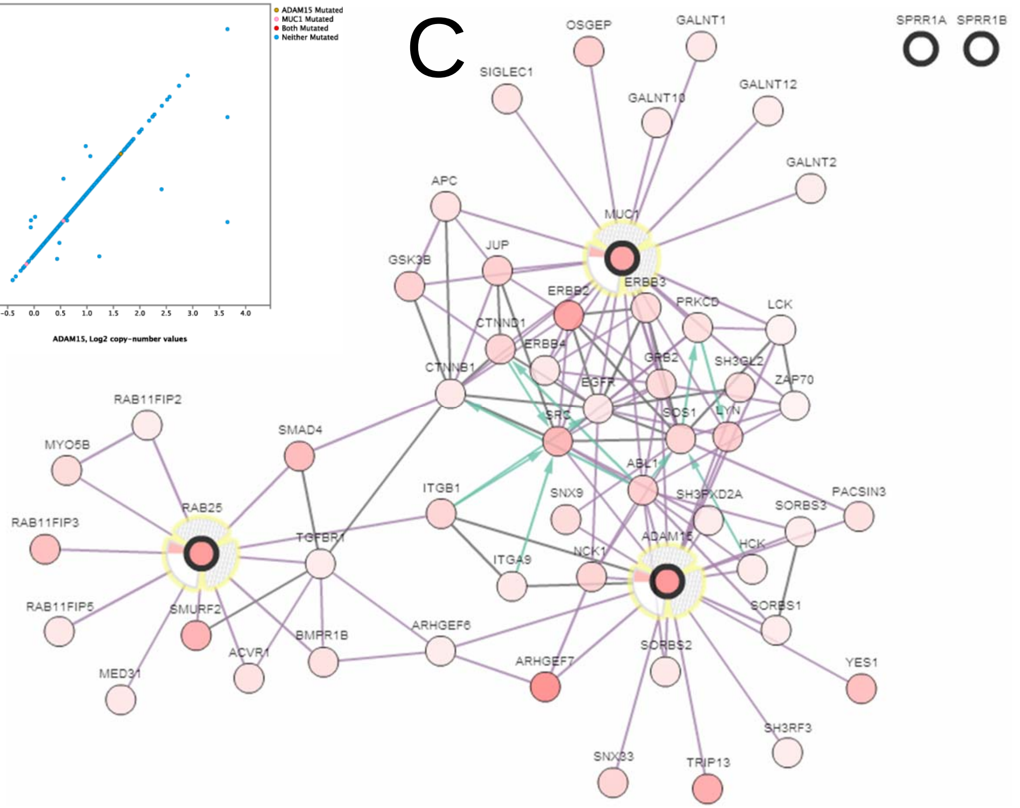

Supplement: Figure S3 — Co-alterations in 5 genes located on chromosome 1. A. Oncoprint generated using cBioPortal of genomic copy number and mutational changes in the members of the 55 Gene Set. B. Two gene comparisons of ADAM15 (x-axis) against RAB25, SPRR1A, and MUC1, respectively. C. Network of interactions of ADAM15, RAB25, SPRR1A, SPRR1B, and MUC1 including the 50 most mutated genes within the network. (PDF) [file pone.0087610.s003.pdf]
